# Overview of Extensively Employed Polymeric Carriers in Solid Dispersion Technology

**DOI:** 10.1208/s12249-020-01849-z

**Published:** 2020-11-08

**Authors:** Athira R. Nair, Yarlagadda Dani Lakshman, Vullendula Sai Krishna Anand, K. S. Navya Sree, Krishnamurthy Bhat, Swapnil J. Dengale

**Affiliations:** Department of Pharmaceutical Quality Assurance, Manipal College of Pharmaceutical Sciences, Manipal Academy of Higher Education, Manipal, 576 104 India

**Keywords:** solid dispersion, polymers, dissolution, solubility, stability

## Abstract

Solid dispersion is the preferred technology to prepare efficacious forms of BCS class-II/IV APIs. To prepare solid dispersions, there exist a wide variety of polymeric carriers with interesting physicochemical and thermochemical characteristics available at the disposal of a formulation scientist. Since the advent of the solid dispersion technology in the early 1960s, there have been more than 5000 scientific papers published in the subject area. This review discusses the polymeric carrier properties of most extensively used polymers PVP, Copovidone, PEG, HPMC, HPMCAS, and Soluplus® in the solid dispersion technology. The literature trends about preparation techniques, dissolution, and stability improvement are analyzed from the Scopus® database to enable a formulator to make an informed choice of polymeric carrier. The stability and extent of dissolution improvement are largely dependent upon the type of polymeric carrier employed to formulate solid dispersions. With the increasing acceptance of transfer dissolution setup in the research community, it is required to evaluate the crystallization/precipitation inhibition potential of polymers under dynamic pH shift conditions. Further, there is a need to develop a regulatory framework which provides definition and complete classification along with necessarily recommended studies to characterize and evaluate solid dispersions.

**Supplementary Information:**

The online version contains supplementary material available at 10.1208/s12249-020-01849-z.

## INTRODUCTION

In the quest to access the complex targets, most of the New Chemical Entities (NCEs) in the development pipeline are becoming increasingly lipophilic, thereby restricting their aqueous solubility. Therefore, a drug candidate with good permeability but limited aqueous solubility is not drug-like due to consequent lower bioavailability(1,2). For such candidates, which belong to Biopharmaceutical Classification class-II & IV (BCS-II & IV), improving the aqueous solubility is the most prudent option. To that end, various techniques have been employed to improve the solubility like micro/nanoparticle drug delivery (3), co-crystal formation (4,5), complexation with cyclodextrins (6,7), and salt formation (8). Nevertheless, success is usually marginal due to the inability of these techniques to generate and sustain the supersaturated state (9,10). In this regard, solid dispersion (SD) is considered as a promising technique, owing to its ability to generate and sustain supersaturation, leading to improved bioavailability (11,12).

**Fig. 1:**
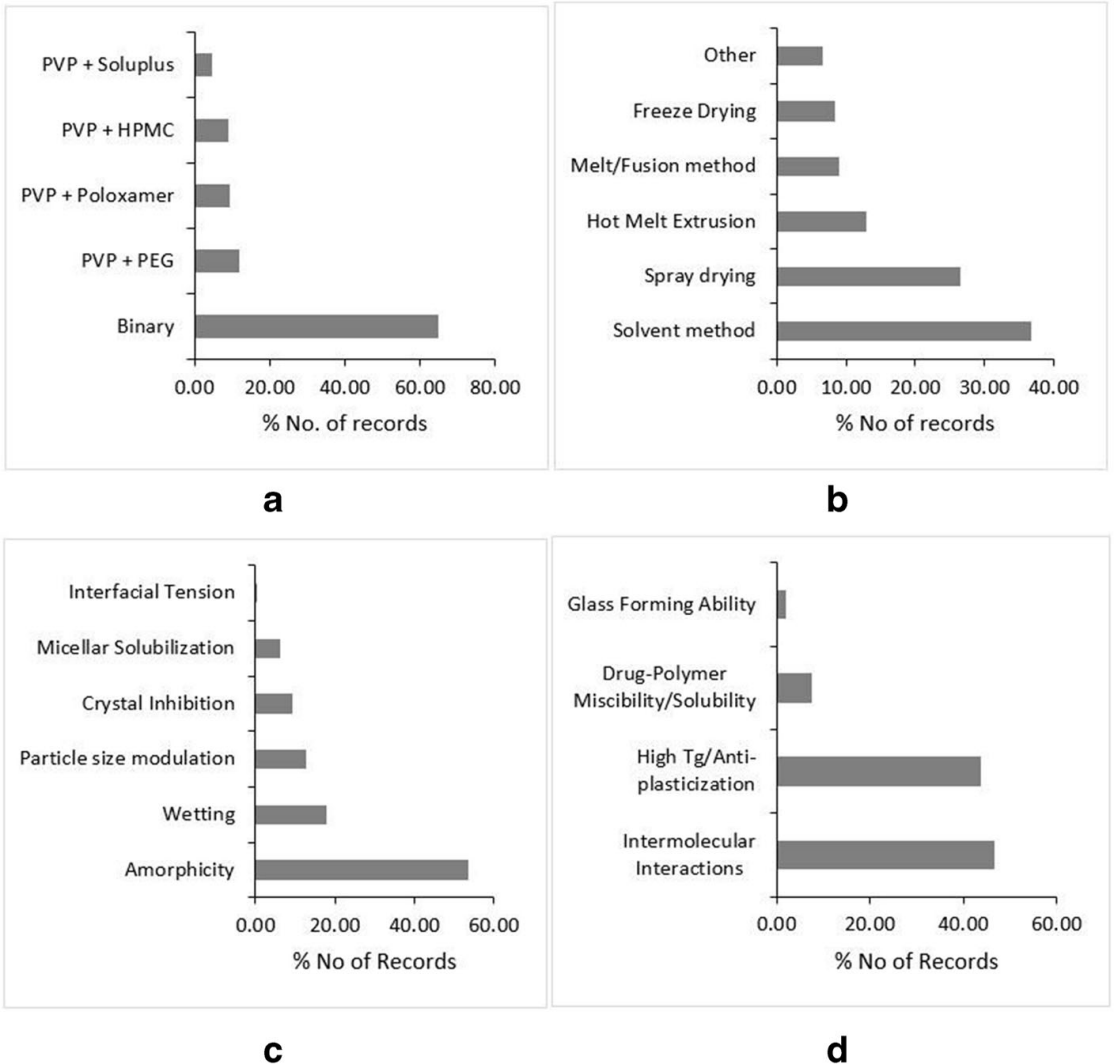
Literature trends of PVP-based dispersions by type of dispersion (**a**), preparation methods (**b**), solubility and dissolution mechanism (**c**), and stability mechanism (**d**). Source: Scopus® database

The first reported solid dispersion attempt was from Sekiguchi and Obi in 1961(13). However, the term solid dispersion was defined by Chiou and Riegelman in 1971 as “a dispersion of one or more active ingredients in an inert carrier at the solid-state prepared by the melting (fusion), solvent, or melting-solvent method” (14). In order to prepare efficacious and stable solid dispersion, the choice of carrier is of great significance. In a solid dispersion, an ideal carrier is required to perform multiple functions (Fig. 1-supplementary material). First, the carrier is expected to improve the solubility and dissolution of the principal drug. This can be achieved by various mechanisms like particle size reduction, improved wettability, and amorphous state of the drug or combination thereof. Second, the carrier is required to impart the physical stability to the prepared dispersion. This chiefly holds well when the physical state of the dispersion is amorphous. The stability to dispersion or amorphous solid dispersion (ASD) is imparted through high glass transition temperature (Tg) of a carrier, intimate mixing, and molecularly dispersed drug in a carrier, intermolecular adhesive interactions or combination thereof (15–21).

Early attempts (1960’s decade) of formulating solid dispersions were focused mainly on using small molecular weight water-soluble crystalline carriers like urea and sugars. Many of these reported solid dispersions belonged to the category of Eutectics. They exhibited excellent thermodynamic stability due to their crystalline nature; nevertheless, the drug release from such formulations was found much slower than amorphous forms (15,22). These limitations of crystalline carriers created an impetus to formulate solid dispersions by employing carriers that have the ability to stabilize drugs in their amorphous state. The space of such carriers has arguably been assumed by polymers, especially amorphous polymers. A solid dispersion prepared by using polymeric carrier falls in the subcategory of solid solutions, glass solutions, or solid suspensions (Table I) (12,14,23). Polymeric carriers have been proven to be the most successful and extensively investigated subgroup. Beginning from mid-2000, there has been a shift in the strategy to formulate solid dispersion, which is driven by two important trends. First, instead of the composite function of solubility and stability improvement in a single carrier, there is new wisdom of using a mixture of polymer and surfactant as a carrier. Wherein, the polymer with high Tg is expected to impart stability, while the solubility and dissolution can be improved by surfactant through increased wettability or micellar formation. Second, the polymers with high glass transition temperature are made pliable to formulate by the fusion method by adding a plasticizer (24,25). In the recent past, however, there is an upward trend in using polymeric surfactants like block copolymers, *i.e.*, Soluplus® and various grades of poloxamers in solid dispersion technology (16,22).

**Table I:** Various Types of Solid Dispersions and Their Relative Potential for Stability and Solubility Improvement

SN.	API state	Carrier state	Phase solubility	SD subtype	Stability	Solubility
1	Crystal	Crystal	No	Solid suspension	+++	++
2	Crystal	Crystal	Yes	Eutectics	+++	+
3	Crystal	Amorphous	No	Solid suspension	+++	++
4	Amorphous	Crystal	No	Solid suspension	+	+++
5	Amorphous	Crystal	Yes	Solid solution	+++	+++
6	Amorphous	Amorphous	No	Glass suspension	+	+++
7	Amorphous	Amorphous	Yes	Glass solution	+++	+++

Part of the classification has been reproduced from the reference (23)

Since 1961, there is prolific literature available on the topic of solid dispersion technology. This is evident by almost 5457 records in the Scopus® database on the topic “Solid Dispersion” OR “Solid Dispersions” as of March 2020. The vast number of polymeric carriers like poly vinyl pyrrolidone (PVP), poly ethylene glycol (PEG), and natural polymers like cellulose derivatives (hydroxypropyl methyl cellulose-HPMC, hydroxy propyl methyl cellulose acetate succinate-HPMCAS, ethyl cellulose-EC) have been actively researched. These polymeric carriers possess interesting physicochemical and thermodynamic properties, which enables their usage in formulating solid dispersions. Aplenty dissolution and stability improvement mechanisms were deduced in the literature for each polymer. Seldom, there exists a single mechanism responsible for dissolution improvement. The same is true for the stability of prepared dispersions. Such numerous and overlapping mechanisms may overwhelm the early carrier researcher and limit his/her comprehension to select an appropriate polymer. In this context, the review article attempts to compile all the existing common knowledge and literature trends of dissolution and stability improvement mechanisms of commonly used polymers in solid dispersion technology to help make an informed decision, in particular for early-career researchers in the field. This was achieved by searching through the Scopus® database. The review will cover polymeric carriers employed in binary solid dispersions; however, the discussion about small molecular weight carriers is out of the scope of this manuscript, as this space is assumed by so-called co-amorphous technology. The interested readers are advised to read many interesting reviews about co-amorphous technology (26,27).

## CLASSIFICATION OF POLYMERIC CARRIERS

There are multiple bases to classify the polymeric carriers employed in the solid dispersion technology, like the physical state, relative hydrophilicity, ionization potential, and source of the carrier (15,16,22,23). Of which, the physical state of polymeric carrier is a very important criterion for classification, as it has direct functional relevance to the dispersion prepared. The polymeric carriers can either be disordered amorphous state or ordered crystalline state. It is widely documented that the crystalline polymeric carriers have limited drug solubility (12,28). Thus, a limited amount of drugs can get molecularly dissolved in the crystalline polymer. If the resultant system is a single-phase, then it is usually classified into a sub-category of solid solutions (12,14,23) (Table I) wherein a limited part of a drug is dissolved in the crystalline carrier. For such systems, oftentimes, the difference between the molecular size of the drug and polymeric carrier is very large. Hence, it is hypothesized that a drug gets dispersed in the interstitial spaces of the polymeric carrier. In particular, such systems are called interstitial solid solutions (12,29). PEG is the representative example of this class (15,30,31). The dispersion consisting of amorphous polymeric carriers has a drug molecularly dissolved or form an amorphous precipitate within the carrier. Amorphous carriers tend to form a single-phase amorphous systems with drugs. The systems can be classified as the single-phase glass solutions (Table I) or colloquially called as amorphous solid dispersion (ASD) (12,14,16,32). Solid dispersions with amorphous carriers usually exhibit higher solubility and dissolution rates due to the high energy amorphous phase of the drug. However, these systems demonstrated to have limited physical stability at room temperature, as the drug can possibly recrystallize within polymeric matrix upon storage. Polymers like PVP, polyvinylpyrrolidone vinyl acetate (PVP VA), and HPMC, which have been extensively used in solid dispersion technology, belong to the class of amorphous polymeric carriers.

**Fig. 2:**
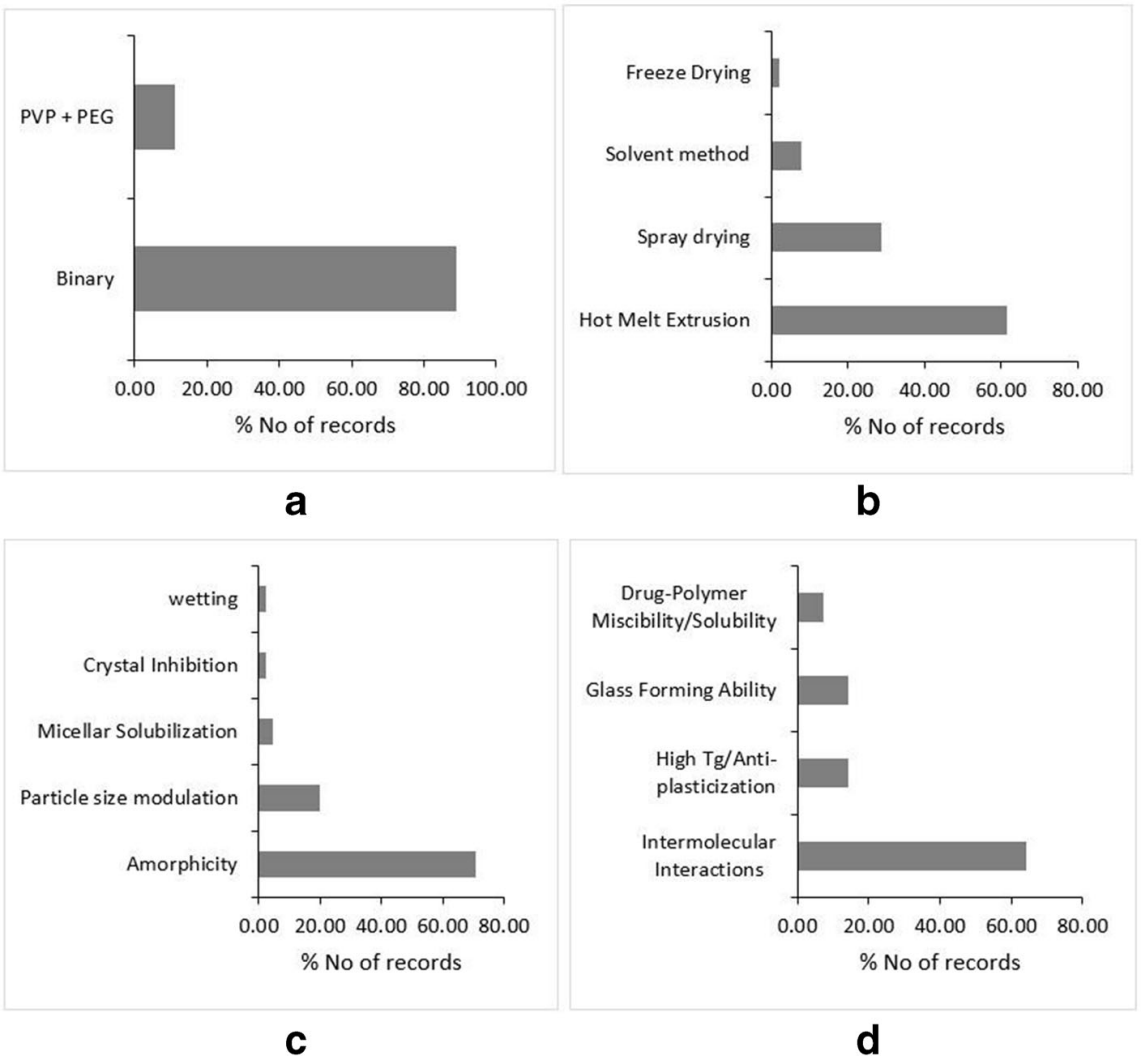
Literature trends of Copovidone-based dispersions by type of dispersion (**a**), preparation methods (**b**), solubility and dissolution mechanism (**c**), and stability mechanism (**d**). Source: Scopus® database

Furthermore, the polymeric carriers can be classified based on their relative hydrophilicity into the categories of hydrophilic, hydrophobic, and amphiphilic polymer-carriers (Fig. 2-supplementary material). The utility of hydrophobic polymeric carriers in solid dispersion technology, which attempts to improve aqueous solubility, seems counterintuitive. However, hydrophobic polymers tend to form excellent single-phase systems with less soluble drugs, which are inherently more lipophilic (BCS-II & IV). The intimately mixed or molecularly dissolved drug within the polymeric carrier is usually inclined to stabilize in the amorphous state and rendering them comparatively more physically stable than hydrophilic counterparts (33–35). The physical stability can be attributed to the excellent phase solubility with drug candidates. If the physical state of the polymer is amorphous, then the phase-soluble systems can be categorized as glass solutions, and phase-separated systems are called glass suspension (Table I). Though the carriers are hydrophobic, the solubility and dissolution improvement is seen in such dispersions due to the amorphous state of the drug (36,37), as the amorphous state does not require the excess energy, compared to the crystalline state, to break down the drug’s crystal lattice in order to solubilize it. Further, hydrophobic carriers are believed to inhibit drug aggregation in solution phase, thus sustaining supersaturation of the drug with respect to its crystalline solubility (37–39). Water-insoluble polymers like cellulose acetate (CA), cellulose acetate phthalate (CAP), ethyl cellulose (EC), methacrylates, and Eudragits fall under hydrophobic polymeric carrier category. Hydrophilic polymeric carriers are by far the most extensively researched topic in solid dispersion technology. If a drug is molecularly dissolved in the hydrophilic polymer carrier, then such type of dispersions are sub-categorized as glass solutions, while phase separated systems are sub-categorized as solid suspensions (Table I) (12,16,40). Though hydrophilic polymeric carriers appear an ideal choice for solid dispersion, being hydrophilic, they exhibit very limited solubility with usually hydrophobic drugs (BCS-II & IV). So, to molecularly dissolve a drug, large quantities of polymers are required (12,41,42). Further, hydrophilic polymers are deliquescent. The limited drug loading in a high proportion of polymer and deliquescent nature of hydrophilic polymers compounds the problem of phase separation, rendering the solid dispersion physically unstable (16,41,43,44). However, hydrophilic polymeric carriers have been demonstrated to be very efficient in solubility and dissolution improvement. Mechanistically, the drug molecule gets released as the hydrophilic carrier dissolves, subsequently forming a supersaturated solution of the drug (45–47). Polymers like PVP, PEG belong to this category. The polymeric surfactants like di-block and tri-block copolymers demonstrate amphiphilic properties. The amphiphilic polymers tend to strike a balance between solubility and stability improvement. The solubility improvement is achieved due to surfactant properties through micellar solubilization and increase in wettability, while the polymeric nature helps to stabilize a drug in a disordered amorphous state. Block copolymers like Soluplus® and various grades of poloxamers are categorized as amphiphilic polymers (48–51). Furthermore, the polymeric carriers are also classified based on their ionization tendencies. Usually, these polymers have pH-dependent solubility and dissolution properties. The interested readers can be advised to follow the literature (88).

There exist other miscellaneous grounds to classify polymeric carriers like ionization tendency and source of polymers. Unlike afore discussed classification, these categories are not functionally relevant and hence not discussed in detail in this review.

## SELECTION CRITERIA FOR POLYMERIC CARRIERS

**Fig. 3:**
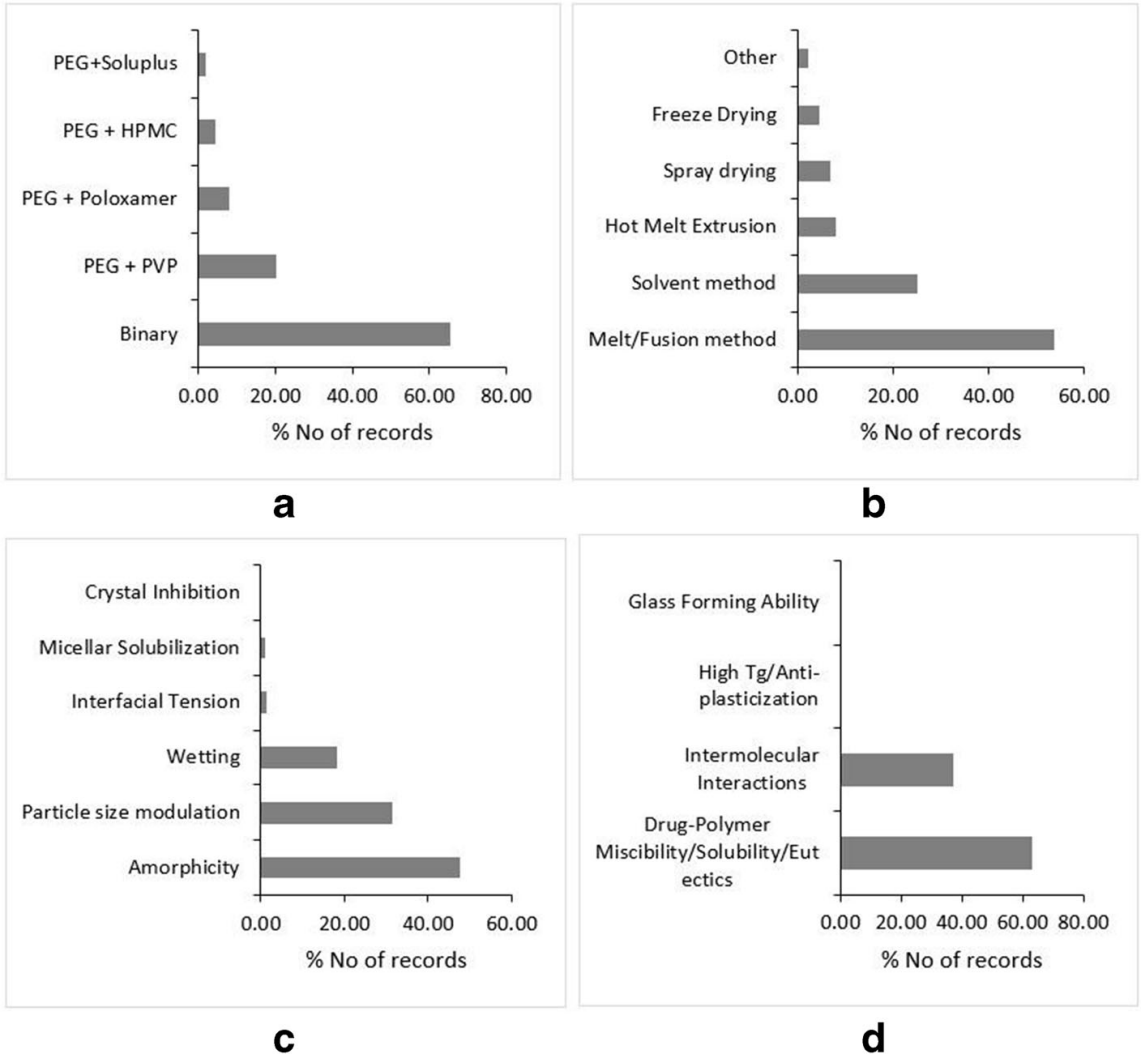
Literature trends of PEG-based dispersions by type of dispersion (**a**), preparation methods (**b**), solubility and dissolution mechanism (**c**), and stability mechanism (**d**). Source: Scopus® database

The desirability of polymeric carriers for preparation of solid dispersion can broadly be influenced by three factors, *i.e.*, safety, kinetic, and thermodynamic aspects (Fig. 3-supplementary material). It is conventional wisdom that any excipient used in pharmaceutical formulations should be inert both chemically and pharmacologically. The same criterion holds good for polymeric carriers employed in the preparation of solid dispersions. The carrier must preferably be Generally Recognized As Safe (GRAS) status (12,22,40,52). From the kinetic perspective, the polymer should be able to inhibit the precipitation of drugs in gastro-intestinal (GI) milieu into their respective crystalline form. Particularly, weakly basic drugs are amenable for precipitation in the intestine due to the predominance of unionized form therein (53,54). Further, if the polymer possesses surfactant properties, then partitioning of the drug in the micelles may help in improving the solubility. Such solubility advantage may be over, and above the solubility, the advantage gained through high energy amorphous state (55–57). The hydrophilic and amorphous polymers are deliquescent in nature. For low load solid dispersions, which usually is a case, the polymer is present in significantly higher proportion, and de-vitrification of amorphous state and subsequent phase-separation pose a significant challenge for physical stability (58,59). Further, thermodynamic factors are important from the stability point of view for dispersions whose physical state is amorphous. The polymer must have a high glass transition temperature (Tg), so prepared dispersion remains stable at room temperature, lowering the probability of Johri-Goldestain (JG) relaxations (20,60–62). If the objective of the formulation scientist is to prepare an amorphous solid dispersion, then the polymer employed must have a good glass-forming ability with a variety of compounds. The high phase-solubility of a polymer with the drug is a pre-requisite to form a single-phase amorphous system (11,52,63). Also, a high probability of adhesive intermolecular interactions through hydrogen bond acceptor and donor properties is desirable to form stable and single-phase amorphous dispersion. From the manufacturing point of view, the solubility of a polymer in organic solvents and phase-solubility aid the preparation of solid dispersion through the solvent and melt method, respectively.

## VINYL PYRROLIDONE DERIVATIVES

**Fig. 4:**
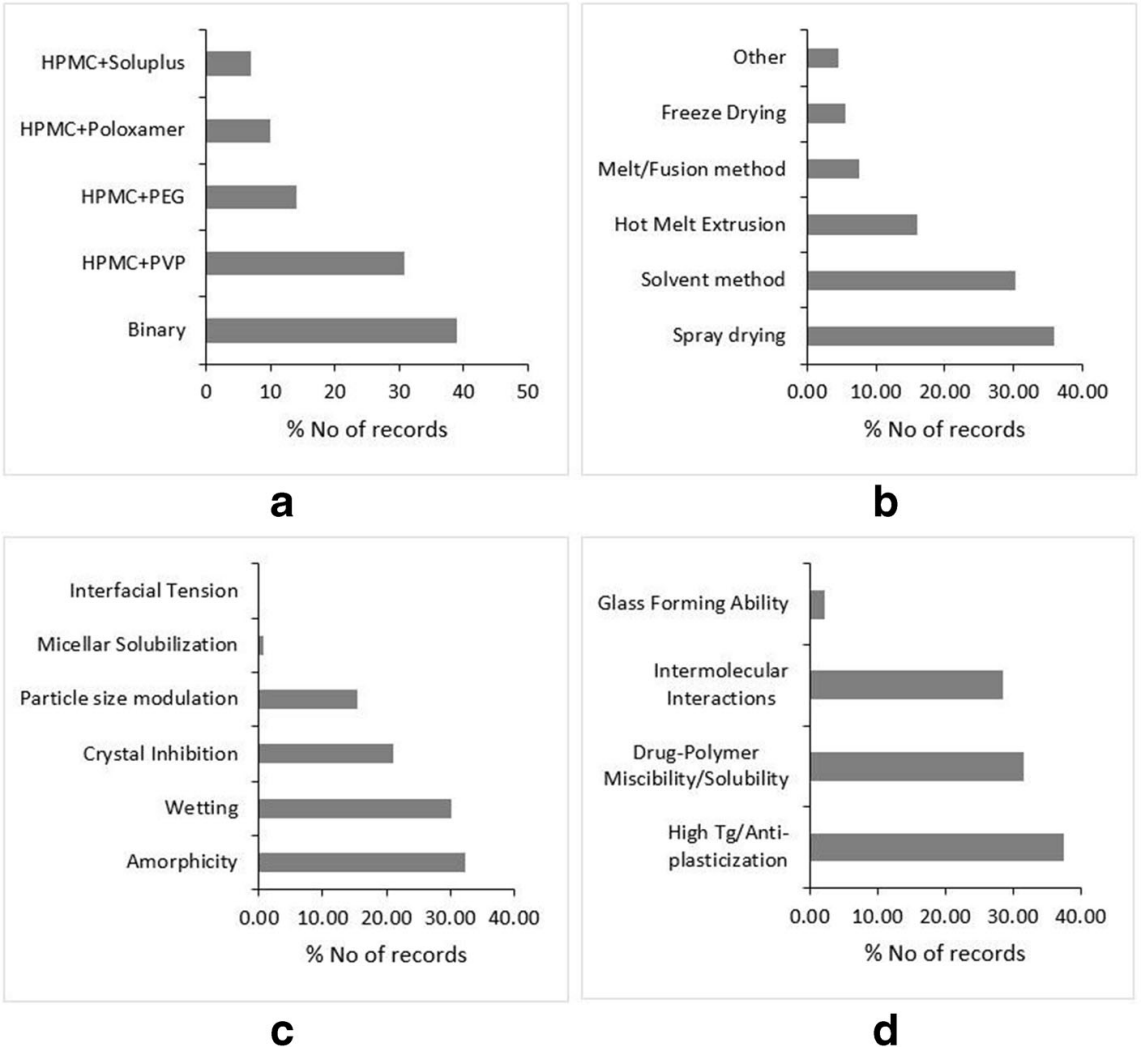
Literature trends of HPMC-based dispersions by type of dispersion (**a**), preparation methods (**b**), solubility and dissolution mechanism (**c**), and stability mechanism (**d**). Source: Scopus® database

The polymerization of N-vinylpyrrolidone yielded a water-soluble product named as polyvinylpyrrolidone and patented in 1939. N-vinyl pyrrolidone derivatives are classified based on crosslinking (Fig. 4-Supplementary material). Since then, PVP has become a widely used excipient in the pharmaceutical industry (64). Further, it was learned that the N-Vinylpyrrolidone undergoes crosslinking at a temperature over 100°C in the presence of alkali hydroxide by the process so-called as popcorn polymerization. The crosslinked product came to be known as Crospovidone demonstrates different physicochemical properties than PVP. The Crospovidone is water-insoluble due to its cross-linkages and hence not widely used in solid dispersions (65,66). Furthermore, to strike a balance between water-soluble PVP and insoluble Crospovidone, the co-polymer of N-vinyl pyrrolidone with vinyl acetate was synthesized which exhibited intermediate polarity between PVP and Crospovidone. The product named as Copovidone is vinyl acetate co-polymer of vinylpyrrolidone in a ratio of 6:4, where 6 parts are vinylpyrrolidone and 4 parts are vinyl acetate. The Copovidone exhibits certain interesting physicochemical properties which enable it to use in formulating solid dispersions (67). The subsequent sections discuss the utility of PVP and Copovidone in the solid dispersion technology.

## POLY VINYL PYRROLIDONE (PVP)

PVP is one of the most commonly used polymeric carriers to formulate solid dispersion. The search through the Scopus database yielded around 939 records of PVP-based solid dispersions from 1978 to date. PVP is commercially available in different grades based on average (mean) molecular weight ranging from 10,000 (10K) to 120,000 (120 K) (Table II) (68,69). The *K* values assigned to various grades of PVP polymer represent a function of the average molecular weight, the degree of polymerization, and the intrinsic viscosity. The *K* values are derived from viscosity measurements and are calculated according to Fikentscher’s formula (Supplementary material). It is a hydrophilic polymer that has an amorphous physical state. It is soluble in water, ethanol, iso-propyl alcohol, and chloroform (16,47).

**Table II:** Physicochemical Properties of Polymers Commonly Used in the Preparation of Solid Dispersions

SN.	Polymer	Physical form	Molecular weight (g/Mol)	Water solubility	Hygroscopicity	Glass transition/Melting temperature (°C)	Degradation temperature (°C)	Solubility parameter (Mpa^1/2^)
1	Poly-Vinyl-Pyrrolidone (PVP)							
1.1	PVP K-12	Amorphous	5000	Water soluble	High	120^a^	196	21.74
1.2	PVP K-15	Amorphous	9700	Water soluble	High	130^a^	215	21.63
1.3	PVP K-30	Amorphous	66,800	Water soluble	High	163^a^	171	21.69
1.4	PVP K-60	Amorphous	396,000	Water soluble	High	170^a^	NA	21.70
1.5	PVP K-90	Amorphous	1,570,000	Water soluble	High	174^a^	194	21.73
1.6	PVP K-120	Amorphous	3,470,000	Water soluble	High	174^a^	194	21.70
2	Copovidone	Amorphous	45,000–70,000	Amphiphilic	Moderate	100–105	230	21.20
3	Poly ethylene glycol (PEG)							
3.1	PEG 400	Crystalline	400	Intermediate	High	NA	NA	18.9
3.2	PEG 600	Crystalline	600	Intermediate	High	NA	160	23.70
3.3	PEG 800	Crystalline	800	Intermediate	High	NA	160	23.70
3.4	PEG 1000	Crystalline	1000	Intermediate	Moderate	37–40	160	23.70
3.5	PEG 1500	Crystalline	1500	Intermediate	Moderate	44–48	160	23.70
3.6	PEG 2000	Crystalline	2000	Intermediate	Moderate	45–50	160	17.6
3.7	PEG 3000	Crystalline	3000	Intermediate	Moderate	48–54	NA	NA
3.8	PEG 4000	Crystalline	4000	Intermediate	Moderate	50–58	NA	NA
3.9	PEG 6000	Crystalline	6000	Intermediate	Moderate	55–63	NA	NA
3.10	PEG 8000	Crystalline	8000	Intermediate	Moderate	60–63	NA	19.8
3.11	PEG 10000	Crystalline	10,000	Intermediate	Moderate	62–65	NA	16.6
3.12	PEG 20000	Crystalline	20,000	Intermediate	Moderate	60–63	NA	NA
4	Hydroxypropyl methylcellulose (HPMC)							
4.1	HPMC-E	Amorphous	85,000–150,000	Water soluble	High	141^c^	NA	29.95^b^
4.2	HPMC-F	Amorphous	85,000–150,000	Water soluble	High	160^c^	240	29.05^b^
4.3	HPMC-K	Amorphous	85,000–150,000	Water soluble	High	172^c^	260	30.57^b^
5	Hydroxypropyl methylcellulose acetate succinate (HPMCAS)							
5.1	HPMCAS L	Amorphous	50,000	Water soluble above pH 5.0	High	119	204	29.10
5.2	HPMCAS M	Amorphous	50,000	Water soluble above pH 5.0	High	120	190	29.10
5.3	HPMCAS H	Amorphous	50,000	Water soluble above pH 5.0	High	122	NA	29.10
6	Soluplus®	Amorphous	118,000	Amphiphilic	Moderate	70	250	19.40

*NA*, not available

^a^Reference (68). ^b^Hansen solubility parameter reported from (124). ^c^DSC data for samples heated from 0 to 230°C at a heating rate of 10°C/min (43), part of the table has been reproduced from reference (88, 172, 173), the molecular weights used in the table are average molecular weights

Owing to the solubility in volatile solvents like alcohol, PVP is a suitable candidate for the preparation of solid dispersion through the solvent method. The solvent method can be scaled-up by using spray drying. Therefore, a large number of studies attempted to formulate PVP-based dispersions through solvent and spray drying methods (Fig. 1a, b). The Tg of PVP is proportional to its molecular weight (Table II). Most of the high molecular weight PVP has usually high glass transition temperature (Tg), one of the highest within the category, which makes it unsuitable for the melt quench method, particularly for low melting point and thermo-labile drugs. Nevertheless, many reports claim the use of the hot melt extrusion (HME) method for preparation (Fig. 1b). Wherein, the majority of these dispersions belong to the ternary category with additional plasticizer employed to formulate the dispersion (Fig. 1a). Furthermore, the rate of dissolution of PVP-based solid dispersion is found highly dependent on the molecular weight of PVP employed to prepare the dispersions. An increase in the molecular weight correlated negatively with the rate of dissolution, as an increase in the molecular weight results in an increase in the viscosity and swelling of PVP within the solution phase (Table-1-supplementary material). This consequently decreases the diffusion of drug molecules from the surface boundaries of the viscous material into the bulk of the solution, leading to retarded dissolution (70). The optimum balance between dissolution rate and polymer grade has led to the prolific utility of PVP 30 K grade, among others, to prepare PVP-based dispersions. The drugs Sulfisoxazole (71), Sulfathiazole (72), Phenytoin (73), Chloramphenicol (74), and furosemide (75) are the examples where the decrease in molecular weight of PVP resulted in the improvement of dissolution rates. Also, the dissolution rate and maintenance of supersaturation are attributed to crystal growth-inhibiting properties of PVP(76). Baghel *et al*. (2018) attributed the extended supersaturation to the reduced crystal growth rates of Dypyridamol and Cinnarazine in the presence of PVP K-30 (77). However, for hydrophilic carriers like PVP, the important dissolution improvement mechanism remains to be the release of molecularly dispersed high energy amorphous drug, which releases as hydrophilic carrier dissolves (Fig. 1c) (78). The reduced particle size is also reported as one of the mechanisms of solubility and dissolution improvement (Fig. 1c). The particle size reduction is achieved by techniques like spray drying, electrospinning, and supercritical aerosol solvent extraction techniques (79–81). However, seldom the reduction in the particle size is alone responsible for dissolution improvement, as it is the interplay between amorphicity and particle size reduction, which achieves the goal of a higher dissolution rate.

As the physical state of PVP is amorphous, it demonstrated to form single-phase glass solutions. For the physical stability of glass solutions, the primary goal is to prevent the phase separation and subsequent recrystallization of a drug within the polymeric matrix. In this regard, the phase solubility/miscibility of a drug in the polymeric carrier is considered as the vital factor in the physical stability of these systems, and evidently, the PVP-based dispersions are no exception for this (Fig. 1d). In addition, the intermolecular interactions are also responsible for physical stability, particularly carbonyl functionality of PVP usually forms hydrogen bonds with weakly basic drugs containing –NH2 and –OH functionalities. The physical stability of Telaprevir-PVP solid dispersion is attributed to the intermolecular hydrogen bonding N-H----O and O-H---O between PVP and Telaprevir (82). Furthermore, the abovementioned stability mechanisms are always supported by higher Tg of PVP (Fig. 1d). Even the compounds, which are fragile glasses with low Tg, also demonstrated to form stable dispersions with PVP due to its high Tg (83,84). Nevertheless, the relative hydrophilicity of PVP makes it a deliquescent compound. Therefore, PVP is amenable for moisture uptake, which may lead to amorphous-amorphous or crystal-amorphous phase separation. Kapourani *et al*., (2019) found that Rivaroxaban solid dispersion with PVP underwent amorphous-amorphous de-mixing and subsequent API recrystallization after significant moisture uptake by PVP (85). Interestingly, Chen *et al*., (2018) reported that though there is a significant risk of moisture-induced amorphous phase separation in PVP-based dispersions, such phase separation does not have a profound impact on drug dissolution of slow-crystallizer compounds (86). Completely withstanding with the report, Milne *et al*., (2015) also showed that high relative humidity and an increase in the temperature did not result in the recrystallization of Zopiclone from PVP-based amorphous dispersion(87). The strengths, weaknesses, opportunities, and threats (SWOTs) analysis of PVP with regard to its implementation in solid dispersions are outlined in Fig. 5 in supplementary material.

## COPOVIDONE

Copovidone is a co-polymer of vinyl-pyrrolidone and vinyl-acetate in a ratio of 6:4, which is also popularly known by trade names Kollidon VA64 (BASF, Germany) and Plasdone S-630 (Ashland, USA). This is amorphous, water-soluble polymer, which has traditionally been used as a binder and film-forming agent in the pharmaceutical industry (67). However, Abbott laboratories successfully repurposed the polymer to formulate the solid dispersion of Lopinavir-Ritonavir combination under the brand name Kaletra (88). Only after the success of Kaletra (after 2010) did the scientific community put the focus on the polymer as a candidate for excipient in solid dispersion technology (Fig. 2a). Copovidone has a significant edge over PVP from the processability perspective. First, owing to the relatively less hydrophilic vinyl acetate substituent, Copovidone is able to exhibit phase solubility with a wide range of APIs having varied polarity values. Second, the Copovidone has a glass transition temperature around 100°C, while degrading at a much higher temperature, *i.e.*, 230°C (67,88,89). This wide temperature window presents an opportunity to employ the polymer to formulate solid dispersions of low as well as high melting temperature APIs alike using hot melt extrusion (HME) technology. Therefore, HME is the most widely used method followed by spray drying for preparing Copovidone-based solid dispersions (Fig. 2b).

Copovidone usually forms a single-phase glassy solutions with APIs. The amorphicity of API within solid dispersion is thought of as a major dissolution improvement mechanism, where the molecularly dispersed amorphous API dissolves along with the water-soluble polymer (Fig. 2c). Taylor *et al*., (2019) concluded that Copovidone accounts for the ideal release of Ledipasvir from the solid dispersion making the process polymer controlled. This generated the supersaturated state of Ledipasvir, even exceeding the amorphous solubility of the drug leading to the formation of a colloidal drug rich phase. Such drug rich phase acts as a reservoir which replenishes the absorbed drug fraction leading to the sustained supersaturation (90). Furthermore, Copovidone-based dispersions are also found to generate nanoparticles during dissolution, contributing to improved solubility and permeability. Harmon *et al*., (2016) demonstrated that Anacetrapib forms nanoparticles during the rapid dissolution of amorphous domains of solid dispersions driven by Copovidone. However, the study employed TPGS as a surfactant to prevent rapid, local drug domain aggregation events (91). Recently, Moseson *et al*., (2020) reported that Copovidone has strong crystal nucleation and growth inhibition properties through polymer adsorption onto the drug crystals. Such mechanism demonstrated to prevent the de-supersaturation of the drug and translated into the sustained parachute in the dissolution profile(92).

As revealed by the literature, intermolecular interactions are reported as a major stabilization mechanism for Copovidone-based solid dispersion (Fig. 2d). Among all types of intermolecular interactions, hydrogen bonding is deduced as a type of supramolecular bond responsible for the physical stability of Copovidone. The carbonyl functionality of both pyrrolidone and vinyl acetate blocks was found promiscuous to form hydrogen bonding. Nevertheless, the recent report claimed that Copovidone tends to form intermolecular halogen bonding. The halogen-containing APIs, namely Clotrimazole, Loratidine, Brotrimazole, and Me-DIBF, were found to form halogen bonds with the amide carbonyl of the vinyl pyrrolidone moiety in Copovidone-based solid dispersions (93). Furthermore, Copovidone has a reasonably high glass transition temperature (Table II). Thus, if it forms a single-phase glass solution with APIs, which is usually the case, then the overall Tg-50°C temperature is expected to have a value higher than the room temperature. The APIs exhibiting low glass transition temperatures like Clotrimazole (Tg 30°C) (94) and Nifedipine (Tg 45°C) (95) are reported to be stable in Copovidone-based solid dispersions. Copovidone is relatively less hygroscopic than PVP. This aids in the storage stability of Copovidone-based dispersions. Sotthivirat *et al*., (2013) analyzed the stability of MK-0364 in Copovidone- and PVP-based solid dispersions under varied temperature and humidity conditions. Unlike PVP-based solid dispersions, no degradation was observed in Copovidone-based solid dispersion. Furthermore, under stress conditions, relatively less crystallinity was observed in MK-0364-Copovidone dispersions (96). However, Copovidone has more moisture uptake potential than Soluplus® and HPMCAS. Kapourani *et al*., (2019) investigated polymeric carrier-based solid dispersions of Rivaroxaban under elevated relative humidity conditions, and found that Copovidone-based dispersion resulted in less physically stable dispersion than its HPMCAS and Soluplus®-based counterparts (85). The SWOT analysis of Copovidone with regard to its implementation in solid dispersions is outlined in Fig. 6-supplementary material.

## POLY ETHYLENE GLYCOL (PEG)

PEG is the polymer of ethylene oxide having a molecular weight in the range from 200 to 300,000 g/mol. The physical state of PEG is dependent on its molecular weight. PEG having a molecular weight below 600 are viscous liquids at room temperature, while the polymer with molecular weights up to 8000 and 20,000 are waxy and dry solids, respectively (16,40,47). PEG is a semi-crystalline polymer with both crystalline and amorphous domains within its structure (97). It has a low melting range from 55 to 68°C and good solubility in water, as well as many volatile organic solvents like methanol, ethanol, and chloroform. The excellent solubility of PEG in volatile solvents makes it a suitable candidate to fabricate solid dispersions by the solvent method (Fig. 3b). The low melting range is advantageous in preparing the solid dispersions of low melting drugs by melt (fusion method). Furthermore, such a low melting characteristic of PEG is uniquely leveraged by certain studies to formulate solid dispersions through combination of solvent-melt method, wherein the drug solution in volatile solvents like methanol/chloroform is added into the molten mass of PEG under constant stirring. The method claimed to stabilize drugs in the microcrystalline state within the PEG matrix (98–100). The cursory look of the published reports on PEG-based polymers reveals that comparatively very few studies have followed the scaled-up approaches for the Solvent method and melt method (Fig. 3b). This may be attributed to the trend in the recent past that the utility of semi-crystalline polymers like PEG to prepare commercial binary solid dispersions is outweighed by the amorphous carriers like PVP, Soluplus®, and advantages thereof. Therefore, crystalline polymer like PEG is largely used in small-experimental-scale mechanistic studies or re-purposed as a plasticizer in conjugation with other polymers like PVP in ternary dispersions or to prepare polymeric nanoparticles (Fig. 3a). Historically, it was hypothesized that the large molecular weight crystalline lamellar polymer like PEG traps small molecular weight drugs within crystalline interstitial spaces of the polymer. Thus, these solid dispersions were considered as interstitial solid solutions (14). However, later it was demonstrated that the molecularly dispersed drug resides in the amorphous domains of PEG(30). Further, PEG also demonstrated to form eutectic mixtures with variety of drugs (Fig. 3d).

Unlike PVP-based dispersions, PEG-based dispersions do not exhibit any consistent correlation between dissolution rate and molecular weight of PEG (47,101). There exists a negative correlation for Tolbutamide (102), Indomethacin (103), Phenylbutazone (103), and Griseofulvin (98), while Furosemide (104) and Papaverine (105) dissolution rates correlate positively with the molecular weight of PEG. However, as the formation of PEG-based solid solutions and eutectics are dictated by drug-polymer composition, dissolution and stability are highly dependent on the drug-polymer ratio. In these cases, the rate of dissolution of polymer dictates the dissolution rate of the drug. Therefore, the dissolution rate increases as a function of weight fraction of polymer within the dispersion. Multitude of drugs like Felodipine (106), Lorazepam (107), and Prednisone (108) demonstrated the increasing dissolution rate with increase in the polymer weight fraction. However, this is not a thumb rule, and the drugs with high glass-forming ability show exactly the opposite trend, where increase in the drug weight fraction leads to the improvement in the dissolution rate. Duong *et al*., (2015) contended that higher drug loading of Indomethacin, a good glass-forming agent, increases the amorphicity of the polymer and inhibiting the crystallization of PEG, leading to the higher dissolution rates (109). Furthermore, the molecularly dispersed drug in microcrystalline or nano-crystalline form within the polymeric matrix also reported as a chief dissolution improvement mechanism for PEG-based dispersions (Fig. 3c).

As the crystalline polymer like PEG is believed to form solid solutions and eutectics, understandably, the phase-solubility/miscibility is a pre-requisite to form thermodynamically stable systems (Fig. 3d). Due to the large difference in the molecular weight of a drug and the polymer, usually, drugs have limited solubility within the polymer, and hence, such systems can best be categorized as monotectics (110). Furthermore, the physical stability of PEG-based dispersions is dictated by the physical state of the drug and polymer in the dispersion, *i.e.*, either PEG/drug is present in crystalline or amorphous form. If a drug and polymer are both present in the crystalline phase, as the case in eutectics or monotectics, then such systems have high physical stability at room temperature (Table I). However, if either one or both the components of binary dispersion are in the amorphous form, then thermodynamic factors such as phase-solubility/separation and subsequent recrystallization of the amorphous phase drive the physical stability of these solid dispersions. The first scenario, where a drug is molecularly dispersed in the amorphous domains of PEG, wherein the recrystallization of a drug is usually observed after storing the dispersion at room temperature. The scenario is described for weak glasses like paracetamol (111), Telmisartan (112), and Celecoxib (113). The second scenario, where the polymer is in amorphous form, herein, it is established that PEG has the potential to vitrify after fusion and subsequent cooling. Dordunoo *et al*., (1997) demonstrated that a significant fraction of PEG stabilized in the amorphous state post-fusion and cooling, albeit the amorphous form reverted to recrystallized state gradually upon storage(114). The same phenomenon was observed for Haloperidol-PEG and Fenofibrate-PEG dispersions, where PEG recrystallized rapidly upon storage (115). Nevertheless, if a drug has a good glass-forming ability, then in such cases, the recrystallization of PEG was found arrested by drugs rendering the dispersions physically stable (Fig. 3d). PEG is relatively less hygroscopic than PVP. However, it is comparable or even more hygroscopic than Soluplus®. The presence of ethoxy and hydroxyl groups in the structure of PEG contributes to the absorption of moisture and forms hydrogen bonds with water molecules (116). Thijs *et al*., (2007) reported that albeit the crystalline nature of PEG may restrict the water uptake, nevertheless, once the hydration shell is formed during the prolonged exposure to higher relative humidity, then the moisture uptake enhances exponentially (117). Baird *et al*., (2010) demonstrated that the deliquescent nature of PEG depends on the molecular weight of the polymer, temperature, and humidity. The moisture sorption ability of the PEG negatively correlates with its molecular weight due to the hydrophobicity of the polymer by an increase in the chain length. It positively correlates with the temperature (116). The behavior of PEG at higher humidity levels depends on its molecular weight, which might lead to recrystallization, cake formation, or formation of viscous liquid (116). Bley *et al*., (2010) observed the dissolution performance of Nifedipine/PEG1500 solid dispersion system was decreased and phase-separated after 6 months of storage. It can be inferred that due to the hygroscopic nature of PEG, there is a high chance of phase separation, and it may affect the dissolution performance as well (118). PEG is being frequently used as hydrogels in preparation of pharmaceutical formulations over a decade. Albeit the popularity, PEG cannot form hydrogels by just adding water. Inevitably, PEGs require crosslinking agents for the formation of networks chemically such as acrylate, methacrylate, vinyl sulfone, maleimide, vinyl ether, and allyl ether. Other techniques involve subjecting PEG to UV radiation or photoinitiators (119,120). PEG hydrogels can be modified according to the need of the formulator. Thus, when crosslinked, PEG can withhold the drug release leading to the controlled release of the formulation. On the contrary, non-linked PEG could burst release suggesting its use in immediate release dosage forms. On the other hand, other polymers mentioned in this review apart from Copovidone are reported to absorb the water and form crosslinking networks resulting in hydrogel formation. This ability of the lot can result in limiting the drug release (41,75,121,122). The possible reason for less physical stability can be attributed to the extremely low glass transition temperature (Tg) of PEG and plasticizer potential thereof. Due to the plasticizing nature of PEG, the final dispersion usually tends to be waxy. To put it in a more technical term, this waxy form is oftentimes found to be supercooled liquid, which has α-relaxations (global mobility) responsible for recrystallization. The SWOT analysis of PEG with regard to its implementation in solid dispersions is outlined in Fig. 7-supplementary material.

Notwithstanding the continued active research interest since 1961, only one commercial product of PEG-based solid dispersions has been marketed so far *viz.* Gris-PEG. The commercial utility of PEG-based solid dispersion is limited by several issues, which include the poor glass-forming ability of the polymer, large variability in physicochemical properties as a function of change in the process parameters, stability issues of polymer and drug, and scale-up and manufacturing challenges (40). The physical state of PEG is crystalline, and it is a poor glass former, which leads to the crystallization of the polymer in addition to the recrystallization of the drug during storage. Furthermore, for fusion methods, the crystallinity of the prepared dispersions is found dependent upon the rate of cooling during processing. Save *et al*. found that the rapid cooling of PEG-4000/6000-Nifedipine melts resulted in the formation of metastable amorphous form, while slow cooling of melts yielded crystalline drug within the dispersion (123). In another study, the same pattern was observed, where slow-cooled Tolbutamide-PEG 6000 solid dispersion exhibited a greater degree of crystallinity as compared to flash-cooled samples. Change in the fusion temperature during the processing of PEG-based solid dispersion was also found to manifest in a variable dissolution rate (124). Such variable physicochemical properties, along with stability issues, were demonstrated to pose a significant challenge during the scale-up of the laboratory processing methods. Furthermore, the plasticizing potential compounded with the poor glass-forming ability of PEG also proves troublesome during formulation development. In particular, the development of tablet dosage form employing a wet granulation step could induce the recrystallization of PEG or drug (125). The low glass transition temperature of PEG leads to the soft and waxy dispersion at room temperature. This translates into the slow dissolution of the dosage form, also complicating the manufacturing of solid dispersion, in particular, compression operations(40).

## CELLULOSE DERIVATIVES

Cellulose derivatives are commonly used polymers in the stabilization of amorphous solid dispersions. Its popularity is because of high molecular weight, inability to get absorbed from the GI tract, strong interaction with the drug molecule, and high Tg. Cellulose is a polysaccharide that consists of linear chains of 1-4-linked β-D-anhydroglucopyranose units in variable length. Since the native form of cellulose is weakly water-soluble because of 40–60% of crystallinity and strong inter- and intramolecular hydrogen bonding between the individual chains in cellulose, chemical modification is carried out *via* etherification or esterification of hydroxyl groups. Ether and ester derivative are water-soluble forms of cellulose. In ether derivatives of cellulose, alkyl/mixed alkyl groups are added in place of hydrogen atoms of hydroxyl groups of repetitive anhydroglucose units. Methylcellulose (MC), ethylcellulose (EC), hydroxypropylcellulose (HPC), hydroxyethyl cellulose (HEC), and hydroxypropyl methylcellulose (HPMC) are commonly used ether derivatives of cellulose. Esterification of cellulose alkyl ethers involves the reaction of ω-carboxyalkanoyl groups with monobenzyl adipoyl, suberoyl, and sebacoyl chlorides, and subsequent benzyl ester hydrogenolysis, to avoid cross-linking. Cellulose derivatives are also used as bonding, coating, stabilizing and film-forming agents, plastic sheets, and emulsion stabilizers in formulations. Cellulose derivatives can be classified based on their water solubility, chemical substituents, and pH responsiveness (Fig. 8-supplementary material). Owing to the availability of multiple cellulosic polymers and extensive scientific records thereof, the discussion under this section will be restricted to the prominently used cellulosic polymer in solid dispersion technology, *i.e.*, HPMC & HPMCAS. The interested readers are advised to follow the rigorous review of the cellulosic polymers (41).

## HYDROXY PROPYL METHYL CELLULOSE (HPMC)

HPMC belongs to the broad category of cellulose-based polymers. Cellulose is ubiquitous in structural components of plants and has extensively been used for various applications as a raw material for over 150 years. The important physicochemical and thermodynamic properties of different grades of HPMC are compiled in Table 2-supplementary material (43,126,127). HPMC is a non-ionic, hydrophilic polymer, which is soluble in water, and most of the organic solvents, including methanol, ethanol, propanol, and dichloromethane. The solubility in volatile organic solvents makes it amenable for formulating solid dispersions through solvent evaporation and its automated scaled-up iteration, *i.e.*, spray drying. In contrast, the aqueous solubility helps to utilize the freeze-drying technique (Fig. 4a, b). Though pure cellulose is semi-crystalline (50–60% crystallinity), HPMC is present in an amorphous state exhibiting a reasonably high glass transition temperature (Tg) of 180°C (Table II) (43). Therefore, for HPMC-based dispersions, fusion methods, including Hot Melt Extrusion, are not methods of choice, certainly not for the low melting APIs. Nonetheless, the fusion methods have been utilized extensively in ternary dispersion, wherein HPMC is being used for its role as a crystal nucleation inhibitor and anti-plasticizer (Fig. 4a).

For HPMC-based dispersions, the predominant mechanism for improved dissolution rate and sustained supersaturation is believed to be the crystallization inhibition potential of HPMC (Fig. 4c). In this regard, HPMC is a more potent precipitation inhibitor than PVP (128). HPMC has proven useful to maintain the supersaturation of weakly basic drugs like Felodipine (129), Itraconazole (130), and Celecoxib(131), which are amenable for precipitation in the intestinal part of the GI tract. Before the disordered API undergoes crystallization in the solution phase, the small nuclei of the crystal build-up in the supersaturated solution, the thermodynamic phenomenon called crystal nucleation. Consequently, it is followed by the kinetic occurrence of the growth of crystals around the crystal nuclei (132). It has been demonstrated by multiple studies that HPMC has a pronounced effect on inhibiting the thermodynamics of nucleation by restricting the mobility of the disordered phase. Xie *et al*., (2016) investigated and found that the inhibitory potential of HPMC on precipitation and solution nucleation of Celecoxib. Nevertheless, the study cautioned that in the presence of seed crystals emanating from the undissolved crystalline drug, it is difficult to prevent crystal growth, particularly at high supersaturation levels (131). It is believed that the intermolecular interactions between API and polymer aid to retard the conversion of the disordered phase into a more ordered crystal nucleus, thus reducing the nucleation rate (Fig. 4c). The hydrogen-bonding interactions between Felodipine and HPMC reduced the nucleation rate by increasing the kinetic barrier to nucleation (129). Chavan *et al*., (2018) also found the additional contribution of hydrogen bonding between API and HPMC towards preventing the crystal nucleation and growth in supersaturated solutions (76). However, the role of high energy amorphous form stabilized within the HPMC matrix in generating the supersaturated solutions cannot be overemphasized. Okada *et al*., (2020) observed that upon dissolution of Nifedipine/HPMC solid dispersion, the Nifedipine undergoes phase separation and dissolves independent of HPMC from amorphous Nifedipine rich phase (133). Furthermore, the unique property of glassy HPMC to swell in contact with water is exploited to extend the release of certain APIs through solid dispersion technology. Puncochova *et al*., (2015) demonstrated that dry glassy HPMC transforms into the wet rubbery state after influx of water. This creates a gel layer, and diffusion of drugs through the gel layer is considered as the rate-limiting step during the dissolution of solid dispersion(121).

The numerous enabling properties regarding the physical stability make HPMC a polymer of choice for commercial manufacturing of solid dispersions, as evident by the fact that more than 50% of the marketed solid dispersion products are HPMC based (41). The vast majority of published literature reported that APIs are generally dispersed molecularly in the amorphous state within the HPMC matrix leading to the formation of glass solutions (Fig. 4c). The HPMC-based glass solutions are rendered physically stable at room temperature due to the high glass transition temperature of HPMC (Fig. 4c). As per the Tg-50 K rule, HPMC prevents the β relaxations from undercooled melts preventing the slow de-vitrification process at room temperature (134), even for fragile glasses with low Tg like Nimodipine (135). Boghra *et al*., (2011) showed that HPMC slowed down the de-vitrification of Irbesartan due to its anti-plasticization potential (136). However, to make use of the anti-plasticization property of HPMC, the drug-polymer solubility/miscibility is the pre-requisite, which forms a single-phase amorphous system (12,137). As HPMC has both hydrophilic (hydroxy) and lipophilic (ether) groups within the structure, it exhibits excellent drug-polymer miscibility/solubility with a wide variety of APIs (41). Tian *et al*., (2016) implemented the fluorescent-based method to investigate the indomethacin-HPMC miscibility. The study reported that the miscibility is highly dependent on relative fraction of a drug within the dispersion. They observed that the dispersion samples with less than 50% drug loading were maintained in amorphous form, while the samples with drug loading higher than 50% crystallized within 15 days (138). The drug-polymer miscibility is also found to be dependent on the preparation technique employed to fabricate solid dispersion. The spray-dried solid dispersions of ABT-102 (model drug)/HPMC exhibited strong drug-polymer miscibility with negative Gibb’s free energy manifesting into the greater extent of melting point depression than solvent evaporated dispersions (139). Furthermore, the intermolecular interactions between drug and polymer also contribute towards the stability of the amorphous phase (Fig. 4d), albeit the contribution of such interactions is relatively lesser than PVP. Felodipine formed intermolecular hydrogen bonds with HPMC, thereby increasing the kinetic barrier to the nucleation rate throughout the storage period, thus preventing the phase separation (129). The physical stability of Resveratrol in HPMC-based solid dispersion was found dependent upon the type and strength of intermolecular interactions between drug and polymer (140). Interestingly, a sizable number of studies contends that the presence of HPMC disturbs the cohesive intramolecular interactions within drug molecules, consequently preventing the crystallization of API. Hormann *et al*., (2018) attributed the stability of Nimodipine/HPMC dispersion to disruption of intramolecular hydrogen bonding within the drug molecules (135). Conversely, the strong intramolecular hydrogen bonding within Curcumin molecules impeded the crystallization inhibition potential of HPMC, preventing the molecular level interaction between the polymer and API (141). Therefore, the contribution of intramolecular hydrogen bonding in prevention of recrystallization within HPMC-based solid dispersion is contended in the literature and it warrants further research on the topic. The SWOTs analysis of HPMC with regard to its implementation in solid dispersions is outlined in Fig. 9-supplementary material.

## HYDROXY PROPYL METHYL CELLULOSE ACETATE SUCCINATE (HPMCAS)

**Fig. 5:**
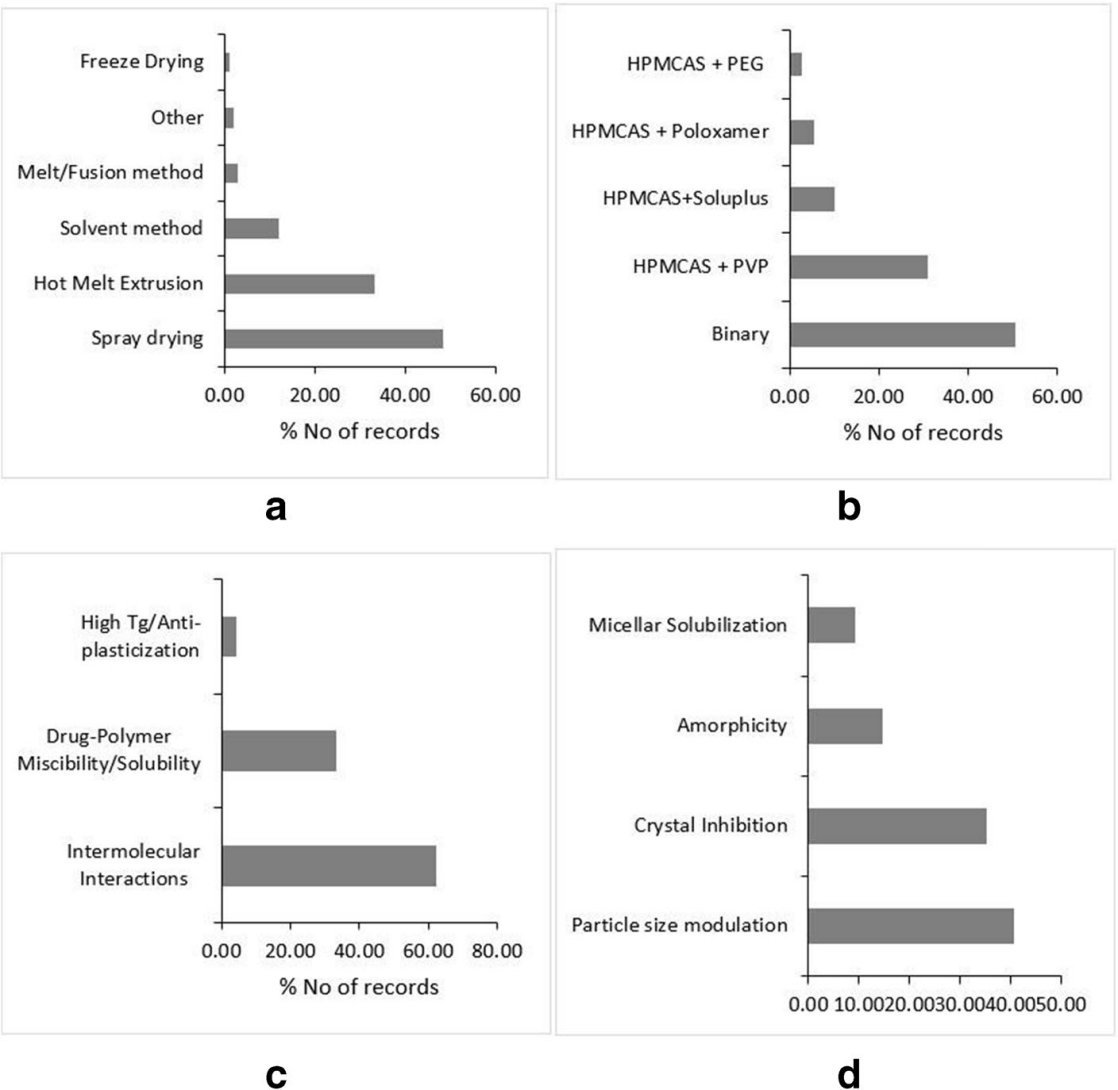
Literature trends of HPMCAS-based dispersions by preparation methods (**a**), type of dispersion (**b**), stability mechanism (**c**), and solubility and dissolution mechanism (**d**). Source: Scopus® database

HPMCAS is a cellulose succinate mixed ester, which is an amorphous amphiphilic derivative of cellulose. Depending upon the acetyl and succinoyl content, HPMCAS is available in three grades, namely L, M, and H (Table 3-supplementary material) (88). Owing to the presence of a succinate group within the structure, HPMCAS exhibits ionization potential. The succinate group within HPMCAS structure has a pKa value 5.0; therefore, the polymer is predominantly unionized below pH 4.0 and ionized above pH 6.0. This ionization behavior manifests in the pH-dependent solubility of the polymer (41,142). HPMCAS is amphiphilic, stable at high temperature, and soluble in organic solvents. These properties put together make spray drying a preferred choice to manufacture HMPCAS-based solid dispersions (Fig. 5a). Further, the large temperature window between the glass transition (120°C) and degradation temperature (270°C) (270–120 = 150°C) is also plentifully explored to prepare solid dispersions by hot melt extrusion (HME) technology (Table II, Fig. 5a) for both low as well as high melting APIs alike (88).

As mentioned earlier, HPMCAS predominantly remains in the ionized form in the intestinal pH conditions (5.0 to 7.4). However, the presence of lipophilic methoxy and acetate substituents limits the solubility of the polymer at pH values above 5.0. Hence, once the solubility limit is exceeded, HPMCAS forms the colloidal phase of Nano-dimension in the intestinal pH conditions, and the negatively charged succinate group keeps the *in situ* nanostructure stable (143). Such nanostructures are found to dissolve rapidly, leading to a faster dissolution rate (Fig. 5d). Much like HPMC, HPMCAS also exhibits recrystallization inhibition properties. Such recrystallization inhibition is believed to propagate through drug-polymer intermolecular interactions and surface adsorption of the polymer on an API crystal seed (Fig. 5c). The drug-polymer interactions in the solution phase are found responsible for recrystallization inhibition for Nimodipine (144), Carbamazepine, and Phenytoin (145), thereby enhancing the solubility and dissolution rate. Albeit, Ueda *et al*., (2014) put forth an alternate narrative about crystallization inhibition potential of HPMCAS. The group demonstrated that HPMCAS suppresses crystallization of Carbamazepine, Nifedipine, Mefenamic acid, and Dexamethasone through molecular level hydrophobic interactions between the drug and polymer(146). Kapourani *et al*., (2019) demonstrated that during crystal growth of Rivaroxaban, small API crystals adhere to the polymer surface due to hydrogen bonding interactions, consequently inhibiting the crystal growth and translating into the improved dissolution (85).

HPMCAS has a higher propensity to form drug-polymer interactions than HPMC. Such intermolecular interactions are responsible for preventing the phase separation in the solid-state, which renders the solid dispersion physically stable (Fig. 5c). Ueda *et al*., (2020) evaluated the intermolecular interactions between Carbamazepine and HPMCAS by employing _1_D -^1^H NMR spectroscopy and found that the mobility of Carbamazepine was strongly suppressed in the presence of HPMCAS due to intermolecular interactions (39). Huang *et al*., (2017) applied coarse grain simulation approach to study the interactions between Phenytoin and HPMCAS and demonstrated that the drug and polymer form a complex due to strong intermolecular interactions. Interestingly though, the protonated polymer chains were found more effective than deprotonated ones in inhibiting the recrystallization of the drug (147). Like HMPC, HPMCAS also has a reasonably high Tg (Table II), which can aid in physical stability. Nevertheless, there are very few reports claiming high Tg of HPMCAS as the sole reason for stability (148). Part of the reason can be attributed to the fact that unlike HPMC, the promiscuous ability of HPMCAS to form intermolecular interactions always confound in many HPMCAS-based solid dispersions as a stability mechanism. Furthermore, the presence of lipophilic substituents over a hydrophilic cellulose backbone gives the wider solubility window to HPMCAS. Therefore, HPMCAS has an ability to be soluble in APIs with varied logP values, and such drug-polymer solubility/miscibility is reported as a physical stability mechanism for HPMCAS-based solid dispersions. The SWOT analysis of HPMCAS with regard to its implementation in solid dispersions is outlined in Fig. 10-supplementary material.

## SOLUPLUS®

**Fig. 6:**
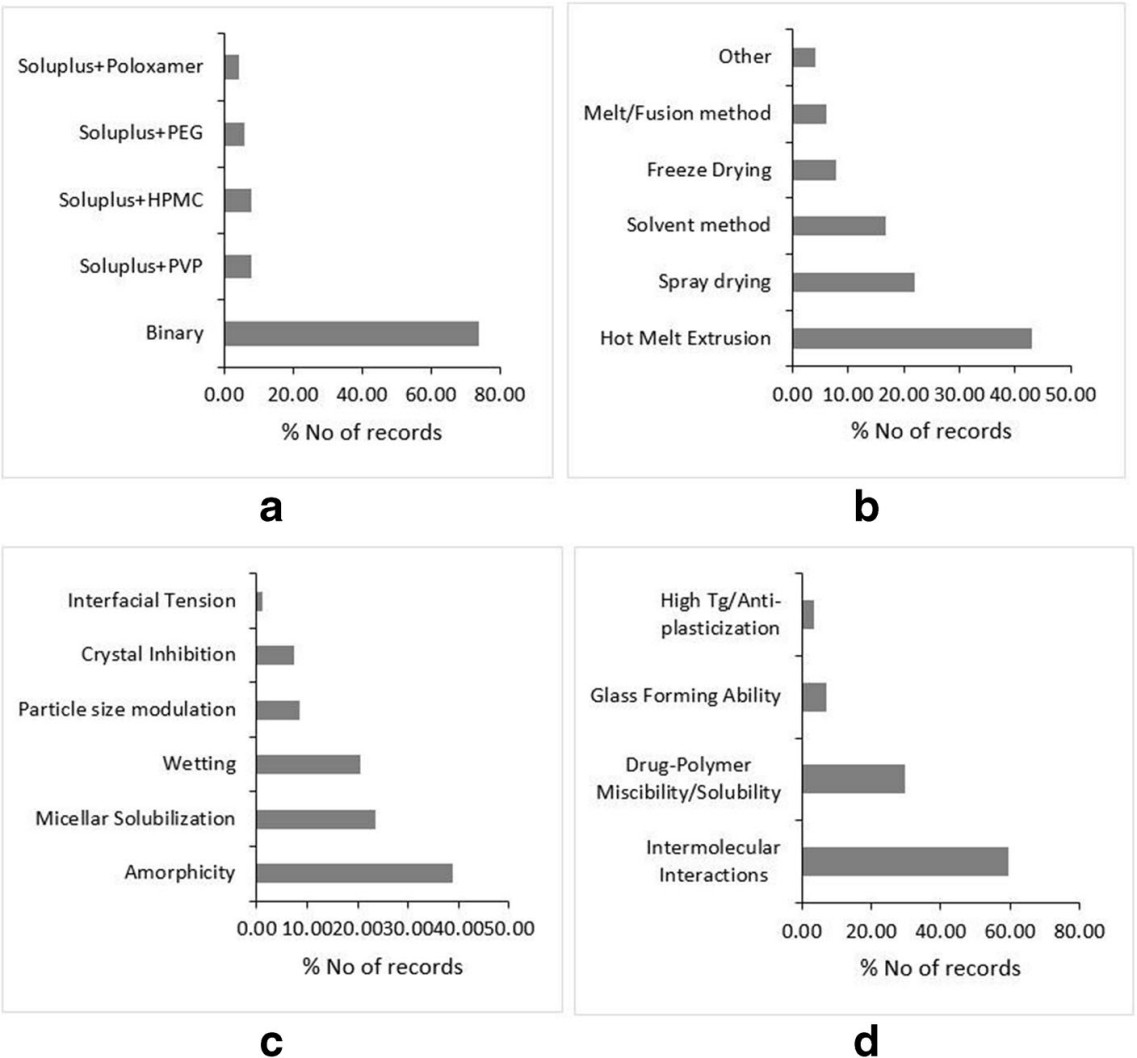
Literature trends of Soluplus®-based dispersions by type of dispersion (**a**), preparation methods (**b**), solubility and dissolution mechanism (**c**), and stability mechanism (**d**). Source: Scopus® database

Soluplus® is a triblock graft copolymer consisting of polyethylene glycol (13% PEG 6000), polyvinyl caprolactam (57%), and polyvinyl acetate (30%). It is an amphiphilic polymer, wherein PEG provides hydrophilicity, while vinyl caprolactam and vinyl acetate domains are lipophilic within the polymer matrix. The molecular weight of Soluplus® usually ranges from 90,000 to 1,40,000 g/mol. It is an amorphous polymer with a relatively low glass transition temperature (Tg) 70°C (149,150). Soluplus® was conceptualized and purpose-developed as an excipient amenable for processing through the hot-melt extrusion process (151). Notwithstanding its plasticization potential, Soluplus® is not widely used in ternary solid dispersions in combination with high Tg polymers like PVP and HPMC (Fig. 6a). Due to the potential of Soluplus® to impart the stability to the glass solutions through mechanisms other than high Tg, it is employed in ASDs without any additional polymers. However, limited studies combined Soluplus® with PVP to make the dispersion pliant for hot-melt extrusion process at low temperature, especially for thermolabile APIs (Fig. 6b). On account of amphiphilic property, Soluplus® is soluble in aqueous as well as organic solvents alike. Solubility in volatile organic solvents makes it suitable candidate to formulate dispersions by solvent evaporation and spray drying. Freeze drying is less frequently employed to formulate Soluplus®-based dispersions even though Soluplus® exhibits good aqueous solubility (Fig. 6b).

Soluplus® is a good glass former, and it forms stable glass solutions with a variety of APIs. For Soluplus®-based dispersions, API is generally dispersed molecularly within the polymer matrix (152). Therefore, upon exposure to the solvent or dissolution media, the API forms a supersaturated solution as it dissolves along with the polymer (Fig. 6c) (149). Owing to the high energy of amorphous API in a supersaturated solution, there exists a thermodynamic driving force working upon API molecules, making them undergo nucleation and crystal growth (132). Therefore, the objective of Soluplus®-based solid dispersions is to maintain the supersaturation. This can be achieved by various mechanisms. Prominent among them is the formation of intermolecular interactions between a drug and polymer (Fig. 6c). Song *et al*., (2019) demonstrated that hydrogen bonding interactions between Andrographolide and Soluplus® lead to enhanced interface wetting, consequently leading to improved dissolution rate (153). Griseofulvin also showed remarkable supersaturation from Soluplus®-based dispersion due to inhibition of API recrystallization through stronger intermolecular interactions (154). There are very few reports outlining the crystallization or precipitation inhibition properties of Soluplus® (Fig. 6c). Guan *et al*., (2019) reported that Soluplus® synergistically inhibited crystal nucleation and growth of Lacidipine, leading to the prolonged supersaturation (155). Soluplus® also exhibits swelling property, which may offset the dissolution rate due to limited diffusion through the swelled polymer. Slamova *et al*., (2020) reported that Tadalafil release from Soluplus® dispersion retarded due to swelling of the polymer during dissolution (122). Nevertheless, the swelling property of Soluplus® can be leveraged to formulate delayed release solid dispersions. Furthermore, owing to its amphiphilic property, Soluplus® forms micelles having a hydrophobic core. Such micelles are capable of a solubilizing variety of solutes, thereby contributing to the improved extent and rate of dissolution (Fig. 6c). More often, the micelles formed with Soluplus® are of nano dimensions, where the average particle size could be sub 100 nm. Micellar solubilization synergistically coupled with reduced particle size contributes to the improved dissolution rate of Soluplus®-based dispersions (150). Zeng *et al*., (2017) demonstrated that Scopoletin/Soluplus® dispersion formed drug entrapped Soluplus® micelles with an average size of 59.4 nm after dissolution (156). However, the solubility and dissolution advantage gained through micellar solubilization can offset in the presence of biorelevant media. It is found that the Soluplus® micelles interact with the bile and lecithin micelles in biorelevant media. This interaction leads to the formation of mixed micelles with increased particle size and altered polarity of the microenvironment of micelles. Pinto *et al*., (2020) found that the solubilization capacity of Candesartan Cilexetil from Soluplus®-based dispersion reduced in biorelevant media (57). Furthermore, if a drug has significant food effect through entrapment into the bile and lecithin micelles of biorelevant media, then micellar solubilization advantage gained by Soluplus®-based dispersion usually diminish. Lakshman *et al*., (2020) found that the dissolution advantage of API from Dolutegravir/Soluplus® dispersion in USP phosphate buffer pH 6.8 was found bridged in biorelevant media. The bridging in the dissolution extent was due to the improvement of dissolution of pure Dolutegravir in biorelevant media which is attributed to micellar solubilization of Dolutegravir in the bile and lecithin micelles, and much less due to the retardation of dissolution from solid dispersion (56).

As Soluplus® is a purpose developed to have good extrudability, it shows relatively lower glass transition temperature (Tg). Therefore, seldom, high Tg is attributed to the physical stability of Soluplus®-based dispersions (Fig. 6d). One of the prominent stability mechanisms for Soluplus®-based dispersions is intermolecular interactions (Fig. 6d). The carbonyl functionality of vinyl caprolactam and vinyl acetate is mainly involved in intermolecular interactions, especially hydrogen bond interactions (139). However, structured intermolecular bonding between API and polymer can be proven complicated for the stabilization of the disordered phase as the disruption of these bonding patterns may manifest into the phase separation. Jog *et al*., (2016) demonstrated the physical stability of ABT-102/Soluplus® dispersion as a consequence of strong hydrogen bonding between –C=O function of Soluplus® and –N-H moiety of the drug(139). Singh *et al*., (2016) prepared Itraconazole/Soluplus® solid dispersion through hot-melt extrusion. The dispersion was found stabilized due to hydrogen bonding interactions between API and polymer. However, while attempting to formulate the tablets from prepared dispersion, Soluplus®-rich regions were formed during compression. It was indicated that the disruption of hydrogen bonding leads to phase separation (157). Oftentimes, the hydrogen bonding interactions are complemented by API-polymer solubility/miscibility with regard to the solid state. In fact, higher API-Soluplus® miscibility may be the result of stronger hydrogen bonding there between. The SWOTs analysis of Soluplus® with regard to its implementation in solid dispersions is outlined in Fig. 11-supplementary material.

## MANUFACTURING ISSUES OF POLYMERS IN SOLID DISPERSION TECHNOLOGY

Solid dispersions are usually prepared by solvent, fusion, or solvent-fusion methods (14). At the manufacturing scale, spray drying constitutes a principal scaled-up version of solvent methods, while the space of fusion methods is unarguably assumed by hot melt extrusion (HME) technology. These techniques have been extensively employed to commercially manufacture various solid dispersion products (Table-4-supplementary material), owing, in part, to their scalability and continuous capabilities (158,159). However, these technologies also have their own set of challenges. Oftentimes, such challenges are dependent on the physicochemical properties of specific polymers, as much they are encountered due to the inherent complexity of the technology. This section discusses the polymer dependent challenges/issues of manufacturing technologies, *i.e.*, spray drying and HME.

Spray drying technology offers many advantages with respect to the manufacturing process-ability of solid dispersion, which includes non-requirement of separate drying and deep-cooling steps, process-ability of thermo-labile compounds, control on the particle morphology, to mention a few (159,160). However, hygroscopic polymer like PVP may lead to amorphous-amorphous phase separation during or after processing. Paudel *et al*. reported the highest residual solvent content in Naproxen-PVP solid dispersion at lower inlet temperature and airflow, while phase separation was observed in response to increased inlet temperature and airflow rate (161). If not instantaneously during the processing, the relatively high residual solvent post-processing can consequently affect the storage stability of the dispersion. Felodipine-PVP solid dispersion was not stable upon exposure to 40°C/75%RH conditions for 8 weeks (162). The issue of residual solvents can be addressed by adding secondary drying unit operation, which employs vacuum dryer or convection tray dryers. Nevertheless, such an additional processing step can incur significant production costs and an increase in production time. It must be borne in mind while preparing PVP-based solid dispersions that certain drugs can get degraded in the presence of PVP solutions. Temazepam was found degrading in the presence of a PVP-K-30 solution in a concentration-dependent manner (163). The problems of high residual solvent content and subsequent instability are also observed in cellulose derivative-based solid dispersions prepared through spray drying. In particular, the inlet temperature has emerged as a critical factor for processing polymers, which have a high probability of hydrogen bond formation like HPMC, HPMCAS (159,160). The glass transition temperatures of HPMC-based solid dispersions were found to correlate directly with inlet temperature. Therefore, a small variation in inlet temperature can translate into the inconsistent Tg and subsequent stability of prepared dispersions through spray drying (161). Furthermore, the surface coverage of drug particles of HPMC-based solid dispersion prepared through spry drying was also found significantly higher than the rota-evaporated product. Such preferential accumulation of drug molecules on the particle surface can be detrimental for dissolution (164).

Though HPMCAS-based spray-dried solid dispersions have been projected as a platform technology by many pharmaceutical industries, it faces several challenges. The major challenge for the preparation of HPMCAS-based solid dispersion in the requirement of large volumes of organic solvents. It is difficult to find a common solvent for water-insoluble drugs and water-soluble HPMCAS (143,159). Curatolo *et al*. used 10 g of acetone to dissolve 133 mg of API and 67 mg of HPMCAS (165). This case in point is further elaborated by Solanki *et al*. that it would require 9480 l of acetone to manufacture a batch of solid dispersion containing 100 kg of the API (166). Multiple studies employed organic solvents like acetone, methanol, ethanol, or combination thereof in the drug-organic solvent ratio in the window of 1:40 to 1:75%*w*/*v* (162,167–169). There are two-fold implications of employing higher volumes of organic solvents. First, there is a possibility of a higher amount of residual organic solvent in the product, which could be toxic and thus not accepted by regulatory authorities. Second, employing such high volumes of organic solvents may pose an environmental hazard. Furthermore, it has been established in the literature that some HPMCAS-based solid dispersions prepared by spray drying were not truly single-phase molecularly dissolved dispersions. Connely *et al*. analyzed Incinek™, which is marketed HPMCAS-telaprevir solid dispersion prepared by spray drying and found that the DSC thermogram of the product showed multiple glass transition temperatures and a melting endotherm of the drug. This indicates that the product is a solid suspension containing multiple amorphous phases and a crystalline phase (170).

HME is a widely adopted technology by the pharmaceutical industry to manufacture solid dispersions (Table-4-supplementary material). Touted as a disruptive technology, it has the potential to make a paradigm shift in pharmaceutical research and manufacturing. The technology has various strengths like the ability to continuously manufacture, scalability, high throughput, customizable, and solvent-free nature (158). Regardless of the popularity, HME faces few issues with regard to chemical degradation and viscoelastic properties of the polymers employed for preparing solid dispersion (171). PVP is the choice of polymer to prepare solid dispersions through HME technology, in particular, due to relatively high glass transition temperature and availability of a wider window between glass transition and degradation temperature. Such anti-plasticization property of PVP requires higher processing temperatures to enable the manufacturing of solid dispersion through HME technology. This often proves deleterious for thermo-labile APIs, limiting the utility of HME technology. Furthermore, the extrusion operation is only possible with complex viscosity of neat polymer between 10,000 to 1000 Pas. Beyond 10,000 Pas complex viscosity limits the extrusion processing capabilities, which pushes the torque limitations of extruders. Lower molecular grades of PVP have a narrow processing window, while higher grades of PVP are too viscous to process without exceeding the torque limitations of the extruder. Polymers, in particular, PVP, HPMC, and HPMCAS exhibit relatively high glass transition temperature and a wider temperature window between glass transition and degradation temperature. However, these polymers cannot be extruded below the degradation temperature due to complex viscosity exceeding 10,000 Pas (171–173). The issue can be and have been remedied by using additional plasticizer, which provides a sufficiently wide processing window below the degradation temperature of the polymers.

## CONCLUSION

In this manuscript, extensively used polymers in solid dispersions such as PVP, Copovidone, PEG, HPMC, HPMCAS, and Soluplus® are discussed thoroughly along with the literature trends. Furthermore, to help with the choice of polymer based on API properties such as melting point, intermolecular interactions, glass forming ability, hygroscopicity, and logP, we have put together a discernment table for the above discussed polymers (Table III). The solid dispersion technology has established itself as a novel way to achieve bioavailability improvement, which is evident by a great deal of publications in the subject area. While it has potential to become a platform technology, it is imperative to give the solid dispersion technology a meaning within the ambit of regulations. Especially in the light of the fact that recently USFDA has come up with a draft guideline for co-crystals, it is necessary to develop a regulatory framework which provides definition and complete classification along with necessarily recommended studies to characterize and evaluate solid dispersions.

**Table III:** Discernment Table for Choosing Polymer Based on API Properties

Drug property	Poly-vinyl pyrrolidone	Poly-ethylene glycol	Hydroxy propyl methyl cellulose	Soluplus®	Copovidone	HPMCAS
Melting point						
High	Yes	No	Yes	Yes	Yes	No
Low	No	Yes	No	Yes	Yes	Yes
Intermolecular interactions						
Molecular bonding	Yes	No	Yes	Yes	Yes	Yes
Glass formability						
Rich	Yes	Yes	Yes	Yes	Yes	Yes
Poor	Yes	No	Yes	Yes	Yes	Yes
Hygroscopicity						
High	No	No	No	Yes	Yes	Yes
Low	Yes	Yes	No	Yes	Yes	Yes
Log P (phase solubility/miscibility)						
High	No	No	No	Yes	No	Yes
Low	Yes	Yes	Yes	Yes	Yes	Yes

## Electronic Supplementary Material

ESM 1 (PDF 1461 kb)
