# Supplementary material for: Overview of Extensively Employed Polymeric Carriers in Solid Dispersion Technology
Source: AAPS PharmSciTech. 2020 Nov 8;21(8):309. doi: 10.1208/s12249-020-01849-z (PMC7649155; doi:10.1208/s12249-020-01849-z)
Supplement: Supplementary file 1 — (PDF 1461 kb) [file 12249_2020_1849_MOESM1_ESM.pdf]

Overview of extensively employed polymeric carriers in Solid Dispersion Technology.

*Athira R. Nair[1], Yarlagadda Dani Lakshman[1], Vullendula Sai Krishna Anand[1], K S Navya Sree[1], Krishnamurthy Bhat[1], Swapnil J. Dengale[1]**

[1]Department of Pharmaceutical Quality Assurance, Manipal College of Pharmaceutical Sciences, Manipal Academy of Higher Education, Manipal-576 104.

***Corresponding Author:**

Dr. Swapnil J. Dengale.*, Associate Professor, M. Pharm., Ph.D.,

Address: Manipal College of Pharmaceutical Sciences, Manipal Academy of Higher Education, Manipal -576 104, Karnataka, India.

E-mail- swapnil.jd@manipal.edu

Telephone-+918202922482

Mobile- +91 9916311176

Fax- +918202571998

Table I: Various grades of PVP and relevant properties thereof.

S. no	Polymer Grade	Viscosity mPas	Density (g/mol)[a]	Heat Capacity (J/gm.K)[a]
1	PVP K-12	1.3 – 2.3	1.04	0.37
2	PVP K-15	1.5 – 3.5		
3	PVP K-30	5.5 – 8.5	1.12	0.28
4	PVP K-60	-		
5	PVP K-90	300-700	1.21	0.29
6	PVP K-120	-		

Reference

a Knopp M et al., (2015) [69].

Table II: Various grades of pharmaceutically relevant HPMC and relevant properties thereof.

SN	HPMC Grade[a]	% Methoxy	% Hydroxypropoxy	% Moisture (%)[b]
1	HPMC E (USP 2910)	28 – 30 (29)	7 – 12 (8.5)	3.92
2	HPMC F (USP 2906)	27 – 30 (28)	4 – 7.5 (5.0)	3.98
3	HPMC K (USP 2208)	19 – 24 (22)	7 – 12 (8.1)	4.73

[a] Subtype of respective polymer is 4M representing molecular weight 4000 g/mol [126].

[b] Moisture content measured by employing TGA analysis [43].

Values in the parentheses represent the USP limits.

Table III: Different grades of HPMCAS and its properties

Properties \ HPMCAS Grade	L Grade	M Grade	H Grade
Acetyl Content	5 – 9%	7 – 11%	10 – 14%
Succinoyl Content	14 – 18%	10 – 14%	4 – 8%
Methoxyl Content	20 – 24%	21 – 25%	22 – 26%
Hydroxypropoxy Content	5 – 9%	5 – 9%	6 – 10%

L, M, H grade: Different grades of HPMCAS; each grade is available in 2 different particle size: fine (F) and granular (G).

Reference: AquaSolve™ hypromellose acetate succinate (hydroxypropyl methylcellulose acetate succinate or HPMCAS). https://www.ashland.com/industries/pharmaceutical/oral-solid-dose/aquasolve-hypromellose-acetate-succinate (accessed Jun 14, 2020).

Table IV: List of marketed solid dispersion product containing PEG, PVP, HPMC and HPMCAS

SN.	Trade Name	API	Polymer excipient	Dosage form	Preparation techniques	Year of launch	Manufactured by
**PEG**							
1	Gris-PEG®	Griseofulvin	PEG	Tablet	Melt method	1975	Pedinol Pharmacal Inc.
**PVP**							
1	Cesamet®	Nabilone	PVP	Tablet	Solvent evaporation	1985	Valeant
2	Kaletra®	Ritonavir/ Lopinavir	PVP-VA64	Tablet	HME	2007	Abbott
3	Norvir®	Ritonavir	PVP-VA64	Tablet	HME	2010	Abbott
**HPMC**							
1	Isoptin® ER-E	Verapamil	HPC/HPMC	Tablet	HME	1982	Abbott
2	Nivadil®	Nivaldipine	HPMC	Tablet	HME	1989	Fujisawa
3	Sporanox®	Itraconazole	HPMC	Capsule	Fluid-bed bead layering	1992	Janssen
4	Prograf®	Tacrolimus	HPMC	Capsule	Spray drying	1994	Astellas Pharma Inc.
5	Rezulin®#	Troglitazone	PVP/HPMC	Tablet	N/A	1997	Pfizer
6	Crestor®	Rosuvastatin	HPMC	Tablet	Spray drying	2002	Astra Zeneca
7	Zithromax®	Azithromycin	HPMC	Tablet	HME	2002	Pfizer, Inc
8	Eucreas®	Vildagliptin + Metformin Hydrochloride	HPMC	Tablet	HME	2007	Novartis
9	Intelence®	Etravirine	HPMC	Tablet	Spray drying	2008	Janssen
10	Modigraf®	Tacrolimus	HPMC	Granules for oral suspension	Spray drying	2009	Astellas Pharma Europe B.V.
11	Samsca®	Tolvaptan	HPMC	Tablet	Granulation	2009	Otsuka Pharma
12	Zortress®	Everolimus	HPMC	Tablet	Spray drying	2010	Novartis Pharmaceuticals
13	Onmel®	Itraconazole	HPMC	Tablet	HME	2010	Stiefel
14	Certican®	Everolimus	HPMC	Tablet	HME	2012	Novartis
15	Incivo®	Etravirine	HPMC	Tablet	N/A	2012	Janssen
16	Astragraf XL®	Tacrolimus	HPMC	Capsule	Wet granulation	2013	Astellas Pharma Inc.
17	Isoptin-SRE®	Verapamil	HPC/HPMC	Tablet	HME	2015	AbbVie Inc.
18	Envarsus® LCPTacrolimus	Tacrolimus	Poloxamer/ HPMC	Tablet	Melt dose technology	2015	Veloxis Pharmaceuticals
19	Zepatier®	Elbasvir/ Grazoprevir	TPGS, Copovidone, and HPMC	Tablet	Spray drying	2016	Merck
20	LCP-Tacro®	Tacrolimus	HPMC	Tablet	N/A	N/A	LifeCycle Pharma
**HPMCAS**							
1	Cymbalta®	Duloxetine	HPMCAS	Capsule	N/A	2004	Eli Lilly

SN.	Trade Name	API	Polymer excipient	Dosage form	Preparation techniques	Year of launch	Manufactured by
2	Incivek®	Telaprevir	HPMCAS	Tablet	Spray drying	2011	Vertex Pharmaceuticals
3	Zelboraf®	Vemurafenib	HPMCAS	Tablet	Solvent controlled precipitation	2011	Roche
4	Kalydeco®	Ivacaftor	HPMCAS	Tablet	Spray drying	2012	Vertex Pharmaceuticals
5	Nofaxil®	Posconazole	HPMCAS/ HPC	Tablet	HME	2013	Merck
6	Orkambi®	Lumacaftor/ Ivacaftor	HPMCAS/ SLS	Tablet	Spray drying	2015	Vertex Pharmaceuticals
7	Torcetrapib®#	Torcetrapib	HPMCAS	Tablet	N/A	N/A	Pfizer

#product withdrawn from market.
HME: Hot Melt Extrusion
N/A: Not available
Reference: [88, 41], and Vasconcelos, T., Marques, S., Neves, J. D., & Sarmento, B. (2016). Amorphous solid dispersions: Rational selection of a manufacturing process. Advanced Drug Delivery Reviews, 100, 85-101.

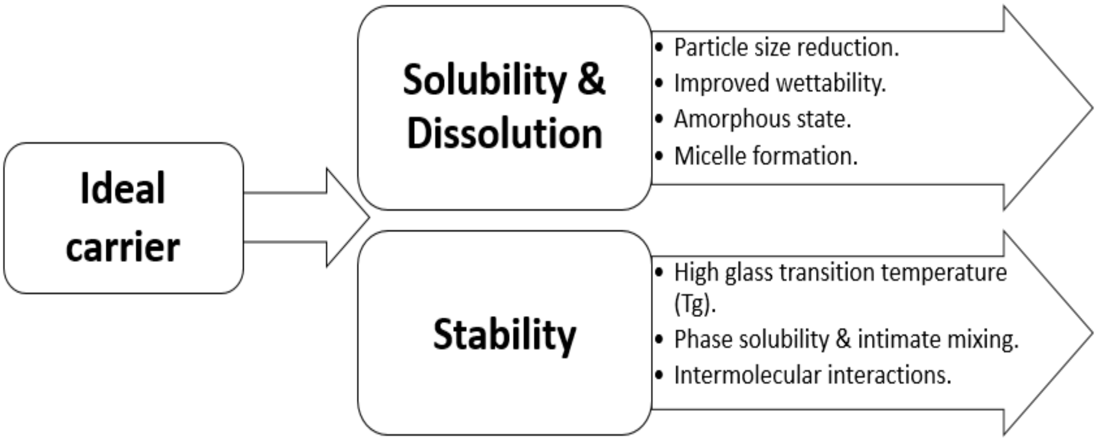

Figure 1: Characteristics of ideal carrier.

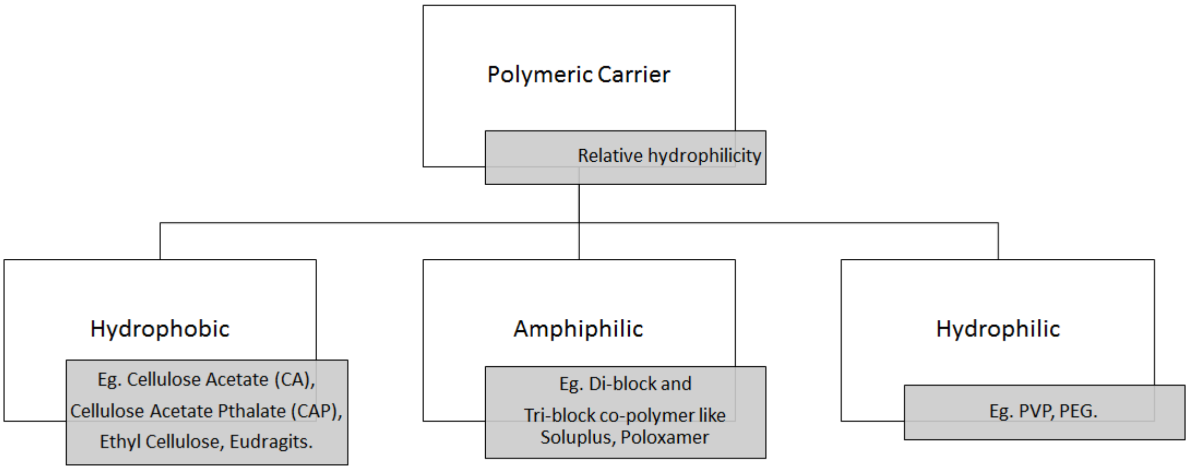

Figure 2: Classification of polymeric carriers based on relative hydrophilicity.

PVP – Polyvinyl Pyrrolidone

PEG – Polyethylene Glycol

HPMC – Hydroxy Propyl Methyl Cellulose

HPMCAS - Hydroxy Propyl Methyl Cellulose acetate succinate

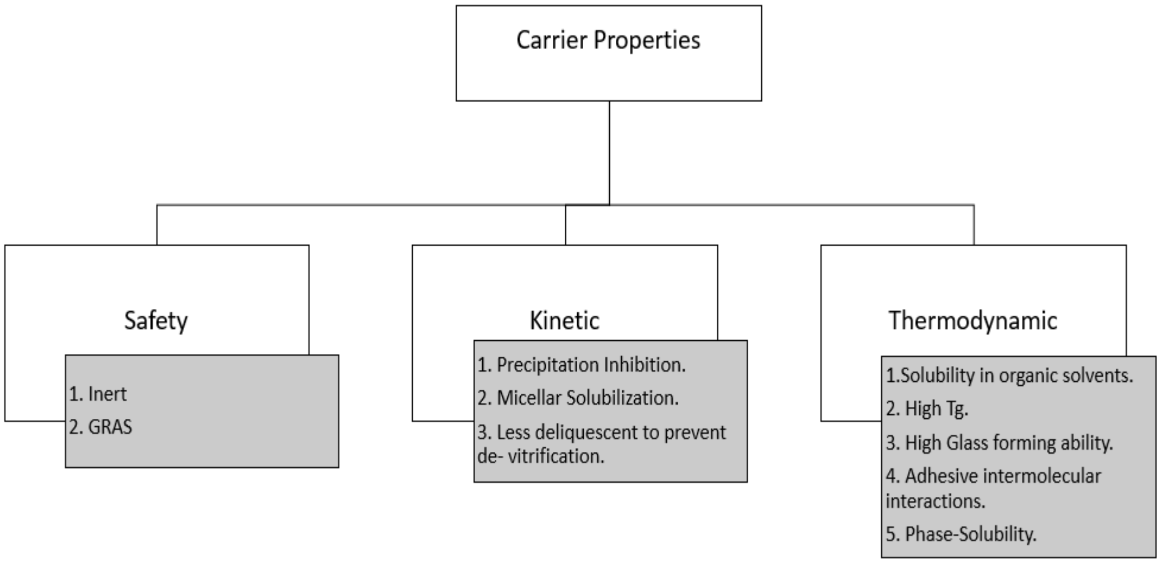

Figure 3: Classification based on carrier properties.

GRAS – Generally Regarded As Safe

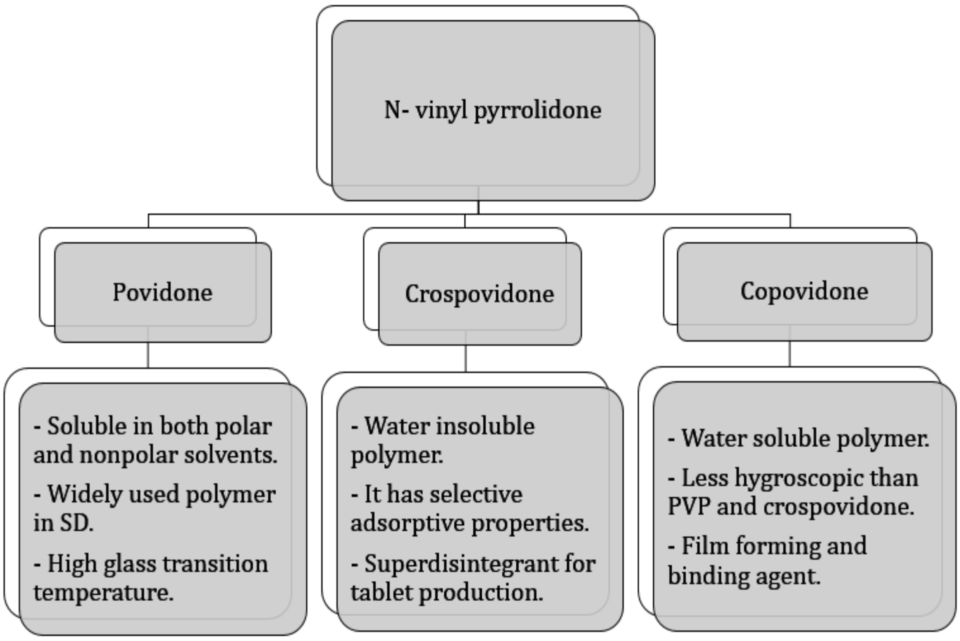

Figure 4: Classification of N- vinyl pyrrolidone based on crosslinking.

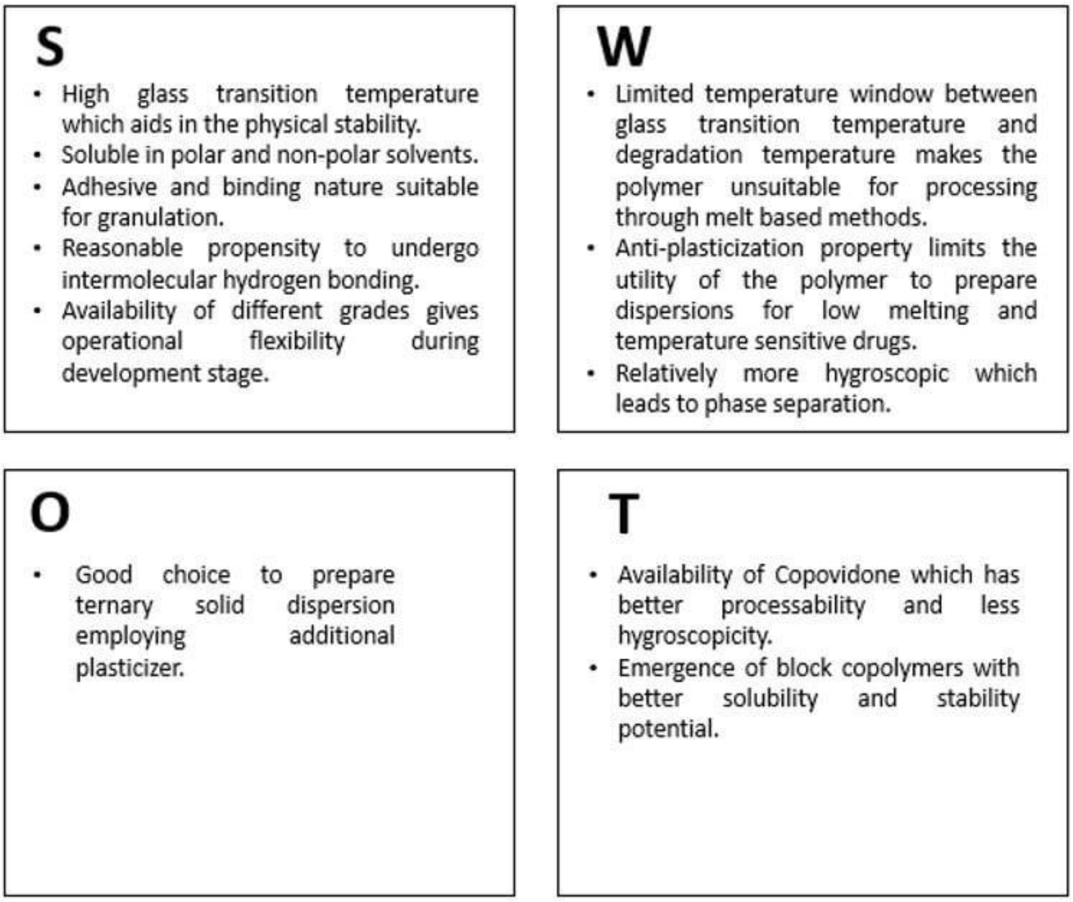

Figure 5: SWOT analysis of PVP with regard to its implementation in solid dispersions.

SWOT – strengths, weaknesses, opportunities, and threats

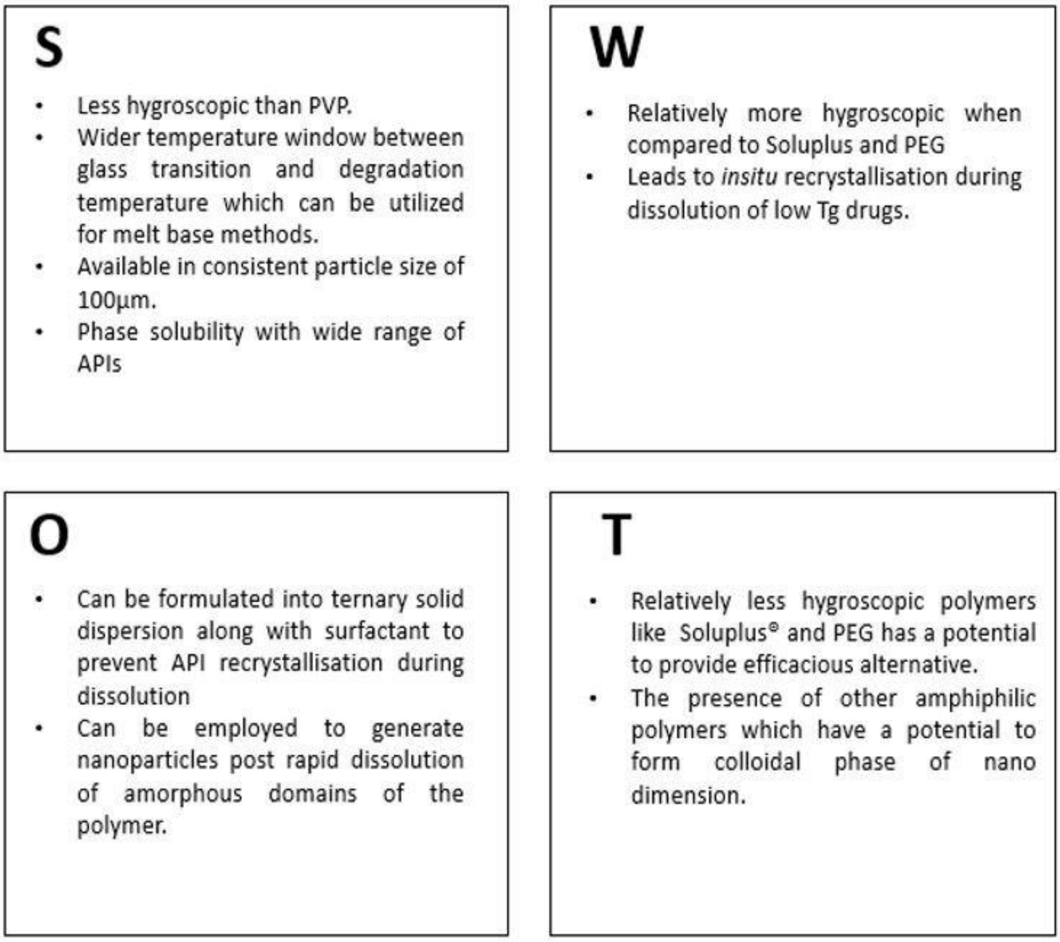

Figure 6: SWOT analysis of Copovidone with regard to its implementation in solid dispersions.

API – Active Pharmaceutical Ingredient

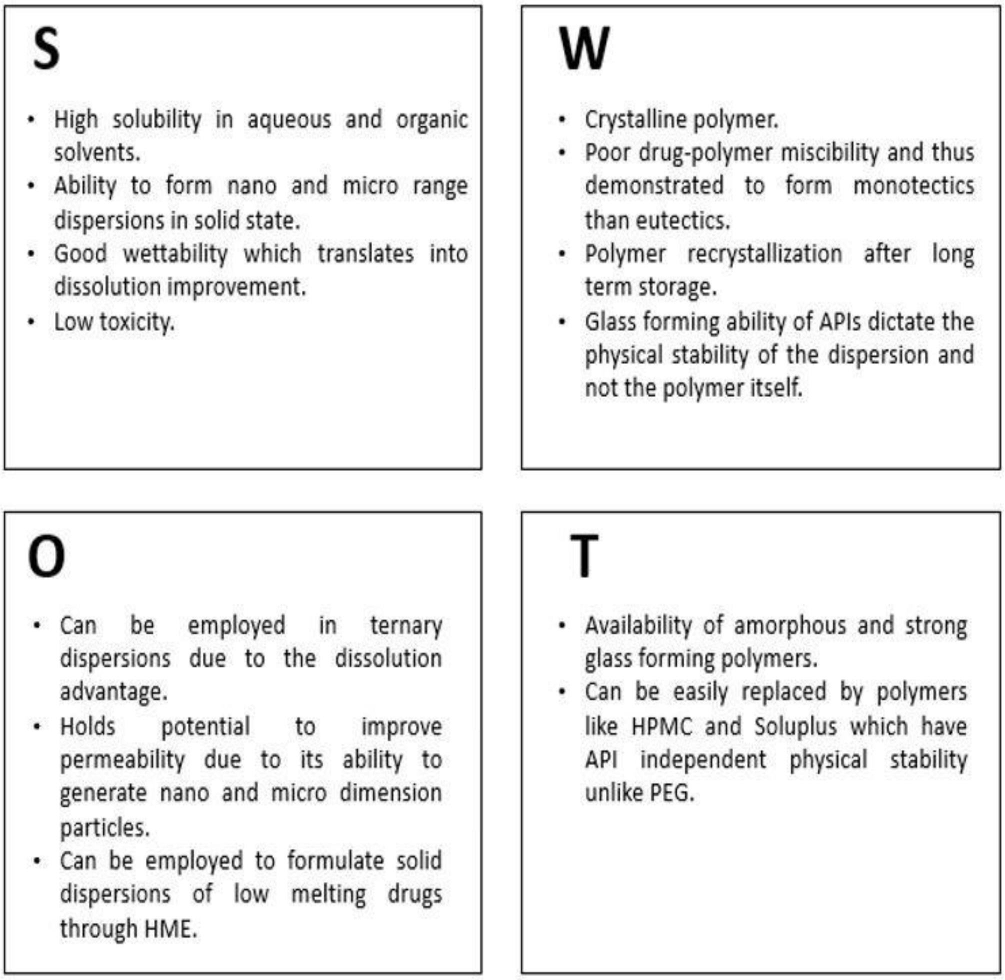

Figure 7: SWOT analysis of PEG with regard to its implementation in solid dispersions.

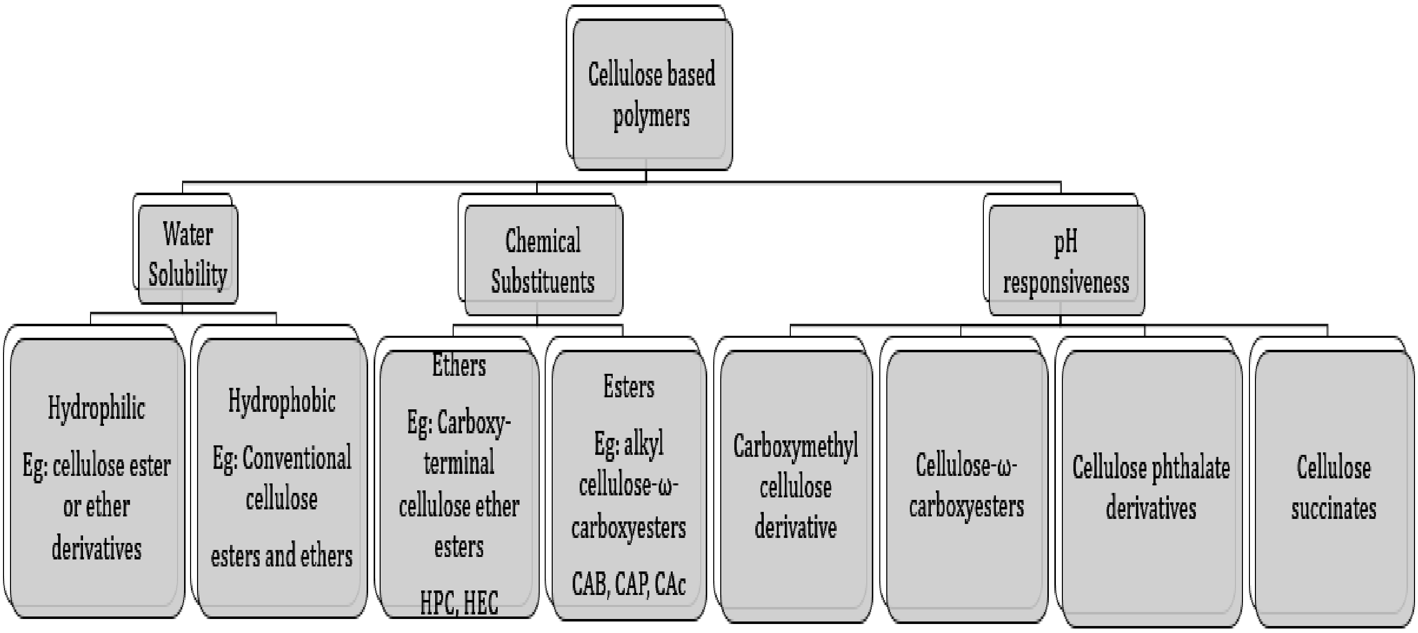

Figure 8: Classification of cellulose based polymer based on water solubility, chemical substituents and pH responsive behaviour. [Part of the classification is reproduced from reference 41]

HPC: Hydroxypropyl cellulose

HEC: Hydroxyethyl cellulose

CAc: Cellulose acetate

CAP: Cellulose acetate-propionate

CAB: Cellulose acetate-butyrate

S

- High glass transition temperature which aids in the physical stability.
- Hydrophilic polymer aids in the dissolution.
- Recrystallization inhibition potential.
- Resistant to hydrolysis and unabsorbed from GIT.
- Excellent biocompatibility and low toxicity.

W

- Poor drug-polymer miscibility for certain lipophilic APIs.
- Unlike HPMCAS, not pH responsive.
- Lacks the ability to form strong intermolecular interactions.
- Limited temperature window between glass transition and degradation temperature of the polymer may limit the utility in melt based preparation methods.

O

- Can be used to formulate ternary dispersions of low melting APIs by using plasticizer.
- Can be implemented to form in-situ nanoparticles post dissolution.
- Can be used to generate sustained supersaturation by the virtue of crystallization inhibition properties of the polymer.

T

- The emergence of polymers like Soluplus which are efficient in dissolution and stability improvement.
- Availability of HPMCAS which is relatively less hygroscopic and pH responsive derivative of cellulose.

Figure 9: SWOT analysis of HPMC with regard to its implementation in solid dispersions.

GIT – Gastrointestinal Tract

S

- Reasonably high glass transition temperature which aids in the physical stability.
- High propensity to form intermolecular hydrogen bonding.
- Strong precipitation /or recrystallization inhibition potential.
- pH dependent solubility and ionization due to presence of succinic acid moiety.
- Availability of different grades gives operational flexibility during development stage.

W

- Limited solubility below pH 4, which may hamper the immediate release of the solid dispersion.
- The method of the polymer synthesis is complicated and inconsistent, which warrants rigorous raw material analysis to prevent batch to batch variability.

O

- Can be used to generate sustained supersaturation and colloidal phase during dissolution.
- Can be leveraged to formulate pH responsive drug delivery systems.

T

- The emergence of polymers like Soluplus which are efficient in dissolution and stability improvement.
- The polymer can be replaced in the future by polymers exhibiting low moisture uptake and high thermal stability.

Figure 10: SWOT analysis of HPMCAS with regard to its implementation in solid dispersions.

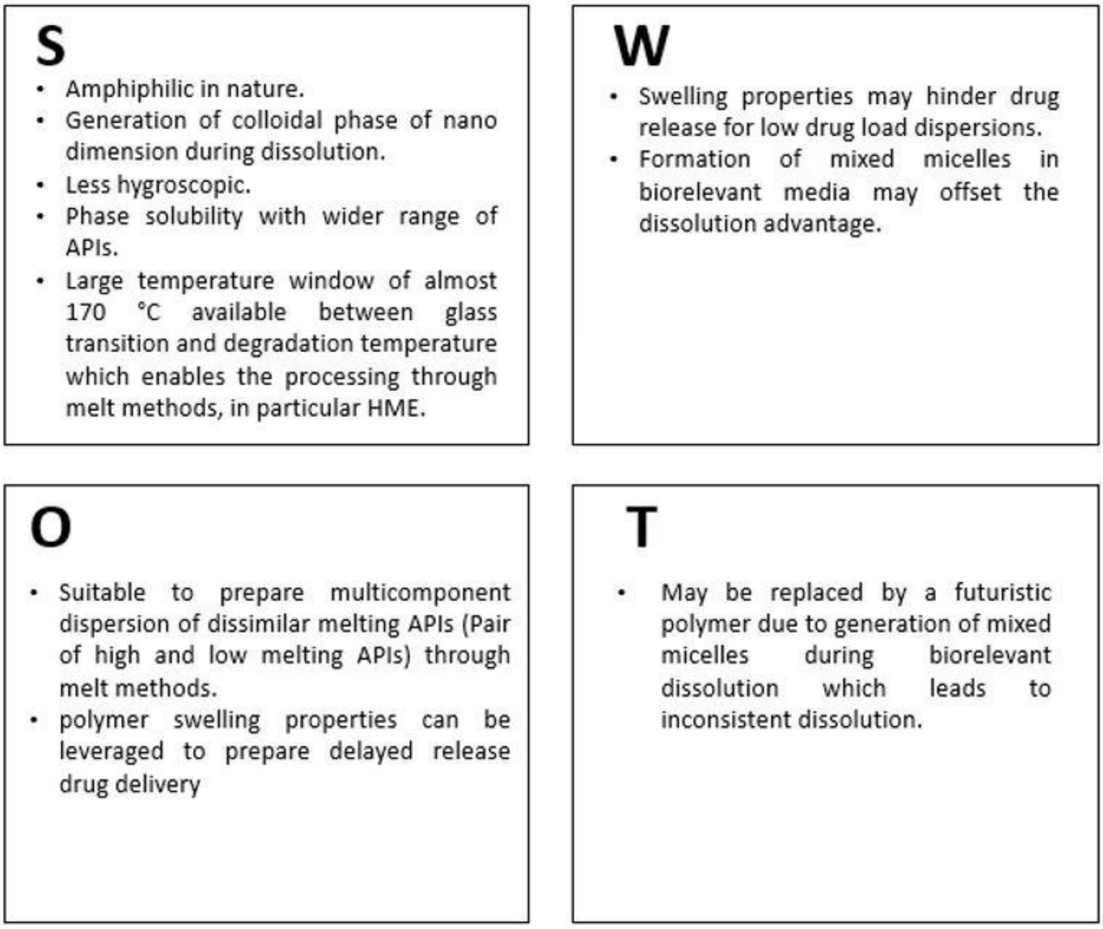

Figure 11: SWOT analysis of Soluplus® with regard to its implementation in solid dispersions.

Fikentscher's k-value:

The K-values assigned to various grades of PVP polymer represents a function of the average molecular weight, the degree of polymerization, and the intrinsic viscosity. The K-values are derived from viscosity measurements and are calculated according to Fikentscher's formula. As a measure of the mean molecular weight of a polymeric substance, Fikentscher derived the k value from measurements of the relative viscosity of solutions of polymer solutions. The k value is calculated by applying the following equation:

$$\frac{\eta_c}{\eta_0} = \left(\frac{75 * k^2}{1 + 1.5 * k * c} + k \right) * c$$

Where:

c = concentration in g/100 ml.
η_c = viscosity of the solution.

η_0 = viscosity of the solvent.
k = value according to Fikentscher (K = 1000* k)
